# Photoreactivity of *E*-*N*,*N*-bis(2-hydroxyethyl)cinnamamide in Methanol and Solid State: A Complementary Experimental and Theoretical Investigation

**DOI:** 10.3390/molecules31193434

**Published:** 2026-09-27

**Authors:** Jacques Kevin Wandji, Sarajit Naskar, Vincent Lemaur, Claudio Palmieri, Amandine Nachtergael, Koen Robeyns, Jérôme Cornil, Rosica Mincheva, Jean-Marie Raquez

**Affiliations:** 1 Laboratory of Polymeric and Composite Materials (LPCM), University of Mons, Place du Parc 20, 7000 Mons, Belgium; 2 WEL Research Institute, Avenue Pasteur 6, 1300 Wavre, Belgium; 3 Laboratory for Chemistry of Novel Materials, University of Mons, Place du Parc 20, 7000 Mons, Belgium; 4 Unit of Therapeutic Chemistry and Pharmacognosy, University of Mons, Place du Parc 20, 7000 Mons, Belgium; 5 Molecular Chemistry, Materials and Catalysis (MOST), Institute of Condensed Matter and Nanosciences (IMCN), Université Catholique de Louvain, 1348 Louvain-la-Neuve, Belgium

**Keywords:** *E*-*N*,*N*-bis(2-hydroxyethyl)cinnamamide (*E*-BHECA), *E* → *Z* photoisomerization, methanolic solution, photodimerization, solid state

## Abstract

*N*,*N*-bis(2-hydroxyethyl)cinnamamide (BHECA) is a photoreactive molecule exclusively known for its selective photodimerization via reversible [2π + 2π] cycloaddition reaction. However, this molecule presents a cinnamoyl core within its structure capable of undergoing reversible [2π + 2π] cycloaddition and/or *E* → *Z* photoisomerization reactions. The present work exploits this hypothesis and provides comprehensive insights into the photoreactivity of *E*-BHECA through complementary experimental and theoretical approaches. Firstly, the impact of the physical state of the reaction medium (methanol vs. solid state (SS)) and irradiation time (six and ten hours) on photochemical outcomes and photoproduct distributions were studied using ^1^H NMR, HPLC-ESI-MS and UV–Vis spectroscopy. Experimental data reveals that in methanol a ten-hour UV irradiation induces selective *E* → *Z* photoisomerization, with a conversion of 49 ± 0.4%. In contrast, irradiation in SS promotes predominant *E* → *Z* photoisomerization (41 ± 0.7%) over minor photodimerization yielding four distinct dimers with unresolved stereochemistry at a very low yield (2 ± 0.3%). Single Crystal X-Ray Diffraction (SC-XRD) reveals an unfavorable γ-crystal form (5.025 Å) of *E*-BHECA that limits photodimerization in the SS. Notably, the major photoproduct, *Z*-BHECA, was isolated and characterized, enabling unambiguous structural and spectroscopic assignments. Secondly, Time-Dependent Density Functional Theory (TD-DFT) simulations of the absorption spectra of BHECA monomers and its possible photodimers confirmed the experimental results, further supporting the predominance of the *E* → *Z* pathway under defined conditions. This study provides additional insights into the photoreactivity of *E*-BHECA, crucial for developing potential applications in pharmaceuticals and smart materials.

## 1. Introduction

Cinnamoyl compounds, characterized by the [(C_6_H_5_-CH=CH-C(=O))-] core, constitute a well-known class of valuable photoreactive species that have been extensively studied and remain of interest [1,2,3,4,5,6]. This is mainly due to their ability to undergo photodimerization via a [2π + 2π] photocycloaddition reaction yielding four-membered cyclobutane structures in a head-to-head and/or head-to-tail configuration, as well as *E* → *Z* photoisomerization under irradiation at a wavelength (*λ*) > 280 nm [5,7,8,9,10,11]. Conversely, upon irradiation at a *λ* < 280 nm, the double bond is reformed through reversible photocleavage of the cyclobutane ring, accompanied by retro-photoisomerization [7,9,12,13]. Interestingly, these photoreactions can proceed without additional compounds such as photocatalysts, photosensitizers, or photoinitiators. Thus, the resulting products contain no traces of unreacted extra dimerization/isomerization agents and their byproducts, which could otherwise limit their applications [6,14]. Additionally, cinnamoyl functionalities have proved useful in dynamic polymer materials, including shape-memory and self-healing materials, photoresists, nanocarriers, liquid crystal display devices, and reversible crosslinked polymers [6,15], important in a multitude of real-life applications and in the conceptualization of a circular economy.

In this respect, *N*,*N*-bis(2-hydroxyethyl)cinnamamide (BHECA) has attracted particular interest, as this multifunctional photoreactive monomer is characterized by the presence of two reactive primary hydroxy groups and a photoreactive cinnamoyl core within its structure capable of undergoing reversible [2π + 2π] cycloaddition and/or *E* → *Z* photoisomerization reactions. Jin et al. reported a more efficient and environmentally friendly route for synthesizing this monomer and demonstrated the exclusive occurrence of the reversible [2π + 2π] cycloaddition reaction upon exposure to alternating UV irradiation in 1,4-dioxane [3]. Moreover, the incorporation of BHECA units into poly(ester)urethanes has been shown to impart light-induced shape-memory effects, arising due to selective photo-reversible [2π + 2π] cycloaddition crosslinking reaction [12,16,17]. To date, photoreactivity studies of BHECA or BHECA-containing polymers have mainly relied on UV–Vis spectroscopy to monitor its selective photo-reversible [2π + 2π] cycloaddition reaction [3,12,16]. Despite these findings, deeper insights into the intrinsic photochemical reactivity of BHECA monomers under various conditions remain underexplored and not yet fully understood. As UV–Vis spectroscopy alone is not a reliable tool for monitoring competing [2π + 2π] cycloaddition and/or *E* → *Z* photoisomerization reactions, employing a broader array of more accurate and sensitive complementary techniques, such as ^1^H NMR [2,11,18,19,20], HPLC, and (HR)MS [20,21], could provide more comprehensive insights on the identity and the distribution of the final photoproducts. Furthermore, parameters such as the crystal form of the starting material, temperature, irradiation wavelength, light intensity, solvent, solvent polarity, and concentration can significantly influence the outcomes and the efficiency of photochemical reactions [5,7,9,20,22]. Additionally, the physical state of the reaction medium (solution or solid state (SS)) and irradiation time also represent key parameters of significant importance in the photochemical reactions of BHECA, but have not yet been addressed in the literature.

To complement experimental investigations and address the current research gap on BHECA’s underexplored photochemical reactivity, computational methods have emerged as a powerful tool to validate experimental results, in order to enhance the grasp of physicochemical processes and molecular behaviors, design superior materials, devices, and drugs [23]. In the present work, TD-DFT was used, owing to its robustness, efficiency, and predictive accuracy [24,25,26], to gain deeper insights on the spectral properties of BHECA monomer and its possible photoproducts which have never been addressed before.

To address the unexplored photochemical reactivity of *E*-BHECA under various parameters, this work presents a dual complementary methodology to gain deeper insights into the photochemical behaviors of *E*-BHECA when subjected to UV light. First, the impact of the physical state of the reaction medium on the photochemical reactions upon exposure to UV is discussed. Specifically, the photochemical outcomes in two reaction media were qualitatively and quantitatively compared: irradiation in a methanolic solution and irradiation in the SS (an environmentally friendly approach that eliminates the use of organic solvents). Second, the influence of irradiation time was evaluated, with the aim of boosting the photoreaction kinetics while minimizing side reactions that could compete with the main photochemical reactions. The findings revealed selective *E* → *Z* photoisomerization in methanol, as well as the predominance of *E* → *Z* photoisomerization over minor photodimerization in SS. Additionally, the major product, *Z*-BHECA was isolated and characterized, enabling unambiguous structural and spectroscopic assignments. Concurrently, TD-DFT studies were employed to further predict the UV–Vis spectra of monomers and possible photodimers complementing the experimental investigations. Notably, the novelty of this study lies not in identifying new cinnamoyl photochemical reactions, but in determining which established photochemical pathway occurs selectively or predominates upon UV irradiation of *E*-BHECA under defined conditions through complementary experimental and theoretical approaches.

## 2. Results and Discussion

### 2.1. Experimental Results

To extend the previous findings and gain deeper insights into the photochemical reactivity of *E*-BHECA, the impact of two key parameters, the physical state of the reaction medium and irradiation time on the outcomes and efficiency of the photochemical reactions, were assessed experimentally. Scheme 1 summarizes their decisive roles in governing the photochemical pathways.

#### 2.1.1. Influence of the Physical State of the Reaction Medium

The investigation of the photochemical reactions that result upon exposure of *E*-BHECA to UV light under various physical states is essential for elucidating the intrinsic molecular transformations of this monomer. In this study, the impact of the physical state of the reaction medium (in methanol and SS) on the photochemical reactions of *E*-BHECA was systematically evaluated using a broader array of complementary ^1^H NMR, HPLC-ESI-MS, and UV–Vis analyses.

In solution, methanol was chosen as the solvent due to its strong solvation ability towards *E*-BHECA, its practical advantages such as affordability, availability, ease of handling, and its UV transparency, which enables efficient photoexcitation. This medium allows greater molecular mobility and less constrained molecular geometries, which often favor *E* → *Z* photoisomerization upon excitation. Additionally, methanol is a polar protic solvent and may influence the electronic excited state responsible for *E* → *Z* photoisomerization through solvent-induced effects such as hydrogen bonding, polarity, dielectric stabilization, and excited-state relaxation phenomena [27,28,29]. Moreover, non-polar solvents fail to efficiently dissolve the monomer, impeding further reliable evaluations. Methanol therefore represents a suitable medium for investigating the photochemical reactions of *E*-BHECA under homogeneous conditions. Conversely, SS medium exhibits restricted molecular motion and geometric/topochemical constraints within the crystal lattice mostly favoring [2π + 2π] photocycloaddition reaction with high regio- and stereoselectivity [1,30]. As a benefit, SS photochemistry eliminates the use of solvents, offering a greener and more sustainable route to the desired photoproducts [31,32].

To investigate the photochemical outcomes of *E*-BHECA, the monomer was subjected to UV irradiation for six hours in methanol and SS. This irradiation time was chosen to give enough time for the photochemical reactions to take place while minimizing the occurrence of side reactions [8,27]. The resulting photoproducts were analyzed via ^1^H NMR spectroscopy and the results are shown in Figure 1 with values reported in Table 1. ^1^H NMR is considered more accurate than UV–Vis and IR spectroscopy for identifying and quantifying photoproducts [11]. It is worth recalling that before UV irradiation, BHECA monomer existed predominantly in the *E*-configuration, as evidenced by a *J* of 15.5 Hz for both H_d_ and H_e_ as revealed by ^1^H NMR analysis (Figure S1 and Table 1).

After six hours of UV irradiation of *E*-BHECA in methanol, the spectrum (Figure 1A, teal) revealed the emergence of two doublet signals at 6.22 and 6.69 ppm, assigned to two new vinylic protons with *J* = 12.8 Hz < *J* of *E*-BHECA = 15.5 Hz. This decrease in *J* from 15.5 Hz to 12.8 Hz indicates the photoisomerization of *E*-BHECA to *Z*-BHECA [2,11,18,20]. Additionally, new peaks were observed: two triplets at 3.54 and 3.59 ppm assigned to the methylene protons in the aliphatic chain, and a multiplet between 7.27 and 7.34 ppm, attributed to the aromatic protons of *Z*-BHECA. However, peaks associated with the protons of *E*-BHECA were still present after six hours of UV irradiation in methanol, indicating a partial photoisomerization of *E*-BHECA.

The degree of *E* → *Z* photoisomerization of *E*-BHECA in methanol after six hours, calculated via ^1^H NMR using [Equation (S2)] was 38 ± 0.4% (Table 1). These results show that six hours of UV irradiation of *E*-BHECA in methanol induce selective *E* → *Z* photoisomerization, in agreement with previous reports on the photoreactivity of cinnamamides in solution [33,34,35,36]. Thus, the *E* → *Z* selectivity in methanol may reflect the combined effect of enhanced molecular mobility and solvent-induced effects. However, the individual contributions of these solvent-induced effects (hydrogen bonding, polarity, dielectric stabilization, and excited-state relaxation) to the photoreactivity of *E*-BHECA in methanol were not assessed in this study.

Additionally, the ^1^H NMR spectrum obtained after six hours of UV irradiation of *E*-BHECA in SS (Figure 1A, black) also showed the emergence of new peaks: two doublet signals at 6.22 and 6.69 ppm with *J* = 12.8 Hz, assigned to the two vinylic protons of *Z*-BHECA; two triplets at 3.54 and 3.59 ppm, attributed to the methylene protons in the aliphatic chain of *Z*-BHECA; and a multiplet between 7.27 and 7.34 ppm, attributed to the aromatic protons of *Z*-BHECA. These ^1^H NMR results indicate the occurrence of *E* → *Z* isomerization after six hours of UV irradiation of *E*-BHECA in SS, in accordance with the literature report on the SS photoreactions of cinnamamide derivatives [37]. Interestingly, two new doublet peaks at 3.97 and 4.04 ppm appeared after six hours of UV irradiation in SS as revealed by ^1^H NMR (Figure 1B, black). These signals were attributed to cyclobutane protons formed by photodimerization via a [2π + 2π] photocycloaddition reaction [2,11,19]. Their absence from the ^1^H NMR spectrum obtained after UV irradiation in methanol (Figure 1B, teal) indicates the efficacy of SS for the [2π + 2π] photocycloaddition reaction related to the compact and regular organization of molecules within the solid structure, which brings the cinnamoyl C=C in close proximity to each other unlike in solution [8,32].

The degrees of photoisomerization and photodimerization of *E*-BHECA in SS after six hours of UV irradiation calculated via ^1^H NMR using [Equations (S4) and (S5)] were 31 ± 0.4% and 1 ± 0.2% respectively (Table 1). These results indicate that *E* → *Z* photoisomerization is the dominant reaction, almost completely suppressing photodimerization, although SS generally restricts isomerization due to reduced mobility. According to the literature, this behavior may be explained by the molecular structure and crystal packing of *E*-BHECA, which might not bring the cinnamoyl C=C in sufficient proximity for efficient photodimerization, while permitting sufficient mobility for *E* → *Z* photoisomerization even in the SS [37]. To confirm this hypothesis, analysis of the crystal structure analysis was performed (see Section 2.1.2). Moreover, the results revealed slower *E* → *Z* photoisomerization kinetics in SS than in methanol due to reduced molecular motion in SS.

To confirm the structural assignment of the major photoproduct formed after six hours of UV irradiation of *E*-BHECA in both methanol and SS, *Z*-BHECA was isolated as a colorless oil and characterized by ^1^H NMR (Figure 1A, red). In contrast, the minor dimer product could not be isolated due to its very low conversion yield (Table 1). The ^1^H NMR spectrum of isolated *Z*-BHECA displayed two doublet peaks at 6.22 and 6.69 ppm, each integrating to one proton with *J* = 12.8 Hz, assigned to the two vinylic protons **e′** and **d′** respectively, of BHECA in the *Z*-configuration. A doublet of triplets appearing at 3.54 and 3.59 ppm was attributed to proton **f′**, and a multiplet between 7.27 and 7.34 ppm was ascribed to protons a′ and b′. In addition, the most deshielded doublet signal in the aromatic region at 7.43 ppm, and the doublet of triplets at 3.62 and 3.78 ppm were assigned to protons c′ and g′ respectively (Figure 1A, red and Scheme 1). Importantly, the chemical shifts and coupling constant of isolated *Z*-BHECA perfectly match those observed in the UV irradiated samples in both methanol and SS.

Furthermore, the thermal stability of the photoproducts generated after six hours of UV irradiation of *E*-BHECA in methanol and SS was also assessed by ^1^H NMR. For this, 30 mg of *E*-BHECA in methanol and SS were exposed to six hours of UV light. Subsequently, the samples were left for two weeks at room temperature in the dark [20]. The ^1^H NMR spectra obtained before and after two weeks at room temperature in the dark were identical for both media, indicating no thermal relaxation to the *E*-configuration under the studied conditions and demonstrating the thermal stability of both *Z*-BHECA and the dimer (Figure S3).

To further confirm the nature of the photoproducts formed after six hours of UV irradiation of *E*-BHECA in methanol and SS, HPLC-ESI-MS, a more sensitive analytical technique was used to complement the ^1^H NMR findings. The HPLC chromatogram of *E*-BHECA before UV exposure is included (Figure 2, blue) to facilitate the identification of new species derived from photoisomerization and photodimerization reactions.

The chromatogram obtained after UV irradiation of *E*-BHECA in methanol (Figure 2, teal) revealed the presence of two peaks: one corresponding to the emergence of a more polar species and the other to unreacted *E*-BHECA at retention times (T_r_) of 14.29 and 16.03 min, respectively. An integrated analysis of the more polar species eluting at a shorter retention time showed an *m*/*z* of 236.0, identical to that of *E*-BHECA (Table 2). The shift to a lower T_r_ with an *m*/*z* of 236.0, corresponding to [BHECA + H]^+^, coupled with the decrease in coupling constant observed in ^1^H NMR, confirms the selective formation of *Z*-BHECA in methanol.

The chromatogram obtained after UV irradiation of *E*-BHECA in the SS is shown in Figure 2, black. Compared with the chromatogram of *E*-BHECA before irradiation (Figure 2, blue), it revealed several new peaks: firstly, like in the methanol, the appearance of a very intense species corresponding to *Z*-BHECA ([BHECA + H]^+^), corroborating the ^1^H NMR findings indicating the occurrence of *E* → *Z* photoisomerization in the SS. Additionally, four new low-intensity peaks labeled *D*_1_, *D*_2_, *D*_3_, and *D*_4_ with T_r_ of 15.37, 18.30, 18.54, and 19.30 min respectively were observed (Figure 2, black and Table 2). The extracted ESI-MS spectra (Table 2) showed that all four species had a higher molecular weight, with equivalent *m*/*z* of 471.1, indicating the formation of four dimer products ([(BHECA)_2_ + H]^+^) with different spatial orientations. Notably, the relative stereochemistry of the four dimers remains unresolved due to their very low conversion yield, which precluded their isolation and structural characterization. However, the HPLC-ESI-MS measurements equally revealed the emergence of two additional very low-intensity peaks (*) at T_r_ of 3.02 and 3.91 min, both with *m*/*z* 261.2. These peaks could indicate the occurrence of side reactions and/or degradation after six hours of UV irradiation of *E*-BHECA in the SS. Notably, corresponding resonance signals attributed to these side products were not detected by ^1^H NMR, indicating that their concentrations remain below the NMR detection threshold.

To further correlate the resulting photochemical reactions after six hours of UV exposure of *E*-BHECA in methanol and SS, UV–Vis absorption spectra were measured (Figure 3). Before UV irradiation, the intense peak centered at 280 nm (Figure 3, blue) corresponds to the dominant π → π* transition of the cinnamoyl C=C of *E*-BHECA [3,12,16]. After six hours of UV irradiation in methanol, the peak decreased in intensity and blue shifted to 274 nm (Figure 3, teal) due to selective *E* → *Z* photoisomerization [38]. Concurrently, the spectrum obtained after six hours of UV irradiation in SS also showed a blue shift to 274 nm, together with a greater decrease in peak intensity (Figure 3, black) related to both predominant *E* → *Z* photoisomerization and minor [2π + 2π] photocycloaddition reaction. It is worth noting that the UV–Vis spectra obtained after UV exposure of *E*-BHECA in both methanol and SS arise from mixtures of photoproducts rather than isolated species. Moreover, the UV–Vis spectrum of isolated *Z*-BHECA exhibited a markedly weaker absorption band centered at 254 nm, corresponding to a diminished π → π* transition of the cinnamoyl C=C (Figure 3, red). This pronounced blue shift and reduced intensity can be attributed to geometric distortion and reduced conjugation between the phenyl ring and the α,β-unsaturated amide fragment in the *Z* structure [39]. Notably, the isolated *Z*-BHECA shows similar spectral changes (decreased intensity and a pronounced blue shift), that have previously been associated with selective [2π + 2π] photocycloaddition reaction of *E*-BHECA [3,12,16]. The present data therefore demonstrate that the alteration of the dominant π → π* transition of the cinnamoyl C=C of *E*-BHECA via UV–Vis spectroscopy cannot by itself unambiguously discriminate between selective [2π + 2π] photocycloaddition, *E* → *Z* photoisomerization or a combination of both competing pathways, underscoring the need for complementary analytical techniques for reliable identification and quantification of photoproduct.

These results demonstrate the impact of the physical state of the reaction medium on the photochemical outcomes. To the best of our knowledge, no previous study has reported the selective occurrence of *E* → *Z* photoisomerization of *E*-BHECA in methanol and in SS, the predominance of *E* → *Z* photoisomerization over photodimerization reaction with very low conversion yields of 1 ± 0.2%. The underlying reason for this very low extent of photodimerization is discussed in Section 2.1.2. In addition, the present study enabled the isolation and characterization of the major product *Z*-BHECA, which has never been reported before.

#### 2.1.2. Influence of Irradiation Time

In this section, the irradiation time was increased from six to ten hours to boost the kinetics of the photoreactions. Specifically, we examined how irradiation time influences the photochemical outcomes and final photoproduct distributions for *E*-BHECA. It is worth noting that due to the high irradiance of the metal-halide bulb (60.649 mW/cm^2^), UV exposure of *E*-BHECA was limited to ten hours in these experiments to minimize side reactions and/or degradation that might compete with the main photochemical reactions of *E*-BHECA in methanol and SS.

Firstly, the photochemical outcome after ten hours of UV irradiation of *E*-BHECA in methanol was assessed by ^1^H NMR. The chemical shifts in the resulting spectrum were similar to those observed after six hours of UV irradiation of *E*-BHECA in methanol, indicating selective *E* → *Z* photoisomerization (Figure 1A, teal).

Table 1 compares the degrees of *E* → *Z* photoisomerization reactions after six and ten hours of UV irradiation of *E*-BHECA in methanol, calculated via ^1^H NMR using [Equation (S2)]. These integrations revealed that increasing the irradiation time from six to ten hours increased the degree of *E* → *Z* photoisomerization to 49 ± 0.4%, owing to a greater number of excited molecules over time. These results demonstrate the impact of irradiation time on the *E* → *Z* photoisomerizability of *E*-BHECA in methanol.

Moreover, HPLC-ESI-MS measurements after ten hours of UV irradiation of *E*-BHECA in methanol also confirmed the ^1^H NMR finding indicating selective *E* → *Z* photoisomerization (Figures S6 and S7 and Table 2).

To further assess the impact of prolonged UV exposure on the photoreactivity of *E*-BHECA, the UV–Vis absorption spectrum of *E*-BHECA in methanol after ten hours of UV light was measured (Figure 4, purple). Compared with the spectrum obtained after six hours of UV irradiation of *E*-BHECA in methanol (Figure 4, teal), increasing the irradiation time to ten hours resulted in a more pronounced blue shift to 266 nm (Figure 4, purple) highlighting enhanced *E* → *Z* photoisomerizability extents, consistent with previous findings and the literature [38].

The impact of ten hours of UV irradiation on the photoreactivity of *E*-BHECA in SS was also investigated by ^1^H NMR spectroscopy and the results are presented in Table 1. The analysis revealed the occurrence of two photoreactions, *E* → *Z* photoisomerization and [2π + 2π] photocycloaddition reactions, consistent with the results obtained after six hours of UV irradiation of *E*-BHECA in SS (Figure 1A, black). The final photoproduct distributions were determined via quantitative ^1^H NMR analysis using [Equations (S3)–(S5)]. The calculated mole fractions of each species are reported in Table 1. The degree of *E* → *Z* photoisomerization increased to 41 ± 0.7% after ten hours of UV exposure. In addition, increasing the irradiation time from six to ten hours had only a limited effect on the photodimerization conversion yield, which increased from 1 ± 0.2% to 2 ± 0.3%. Although these values lie close to the typical uncertainty of quantitative NMR, reproducibility across independent triplicate irradiations and measurements supports the consistent formation of minor dimer products.

To clarify the topochemistry herein, Single Crystal X-Ray Diffraction (SC-XRD) analysis was conducted to evaluate the alignment of adjacent olefinic double bonds in the crystal packing of *E*-BHECA and thereby identify its crystal form. The crystal form is a fundamental parameter in SS photodimerization, which is governed by a number of principles known as Schmidt criteria [4,5,7]. Single crystals were grown by vapor diffusion of pentane into a chloroform solution of *E*-BHECA. The complete crystal data and structure refinement parameters for *E*-BHECA are provided in Table S1, and the resulting structure is shown in Figure S8. The double bonds are aligned in parallel, with a shortest distance of 5.025 Å between the centroids of the double bonds, exceeding the 4.2Å upper limit defined by Schmidt. This unfavorable crystal packing indicates a γ-crystal form of *E*-BHECA, therefore accounting for the very low photodimerization extent in SS, although γ-cinnamoyl compounds are generally photostable. However, the classical Schmidt postulates for SS photodimerization operates under a static ground-state topochemical constraint and is not universally applicable [40]. In this regard, despite of the unfavorable crystal packing of *E*-BHECA (5.025 Å), the minor photodimerization observed in SS may therefore arise from dynamic topochemical features within the crystal lattice. As demonstrated in subsequent studies by Ramamurthy, Venkatesan and co-workers [40,41,42,43], inherent orientational flexibility of molecules, lattice relaxation, overlap of the participating π orbitals, crystal defects and local free volume can transiently bring a small fraction of neighboring reactive moieties into a more favorable geometry following excitation, further rationalizing the occurrence of minor SS photodimerization of *E*-BHECA. Nevertheless, because these dynamic processes were not directly investigated in the present study, their respective contributions cannot be distinguished and remain plausible explanations for the observed minor photodimerization.

To confirm phase purity, the PXRD pattern simulated from the single crystal structure overlaid on the experimental pattern (Figure S9), showed a perfect match.

Figure S10, brown and Table 2 show the HPLC-ESI-MS results after ten hours of UV irradiation of *E*-BHECA in SS. The results revealed the same pattern as seen after six hours of UV irradiation with the emergence of five new peaks: a very intense species at T_r_ of 14.30 min with *m*/*z* 236.0 corresponding to *Z*-BHECA ([BHECA + H]^+^), and four peaks labeled *D*_1_, *D*_2_, *D*_3_, and *D*_4_ with T_r_ of 15.36, 18.29, 18.53, and 19.29 min respectively, all with the same *m*/*z* value of 471.1 corresponding to four dimers ([(BHECA)_2_ + H]^+^) with distinct stereochemistry. These results further confirm the occurrence of two photoreactions after ten hours of UV exposure of *E*-BHECA in SS: predominant *E* → *Z* photoisomerization and restricted [2π + 2π] photocycloaddition reactions. In addition, compared to six hours of UV irradiation, the emerging peaks after ten hours of UV displayed superior intensities suggesting enhanced photoisomerization and photodimerization reactions in accordance with ^1^H NMR findings. Nevertheless, after ten hours of UV irradiation of *E*-BHECA in SS, two additional peaks (*) at T_r_ of 3.02 and 3.91 min, both with *m*/*z* 261.2, showed increased intensity with irradiation time. These peaks suggest enhanced side reactions, reinforcing our strategy to limit the irradiation time to a maximum of ten hours.

Figure 5, brown shows the UV–Vis absorption spectrum of *E*-BHECA after ten hours of UV irradiation in SS. Relative to the spectrum obtained after six hours (Figure 5, black), the absorption maximum further blue shifted to 268 nm (Figure 5, brown) lower than the 266 nm observed after ten hours of UV in methanol (Figure 4, purple), suggesting slower *E* → *Z* photoisomerization kinetics in SS. In addition, a further decrease in the intensity of the absorption maximum was observed after ten hours of UV irradiation in SS (Figure 5, brown) compared with six hours of UV irradiation in SS (Figure 5, black), which may be related to the limited increase in the photodimerization yield.

In the last section of this study, the kinetics of selective *E* → *Z* photoisomerization in methanol were qualitatively assessed via ^1^H NMR (Figure S12). The data revealed rapid *E* → *Z* photoisomerization at shorter irradiation times, followed by a gradual deceleration and more interestingly, the emergence of a plateau after ten hours of UV irradiation, demonstrating that the system reaches a steady photo-stationary state. Notably, prolonged irradiation times (>ten hours) did not significantly increase the degree of *E* → *Z* photoisomerization, further reinforcing our strategy to limit the irradiation time to ten hours.

### 2.2. Quantum-Chemical Calculations

To support the interpretation of the experimentally observed changes in the absorption spectra upon UV irradiation, TD-DFT calculations were performed on the monomers and possible photodimers. The simulations clearly show distinct optical signatures for the *E* and *Z* geometries of the monomer (Figure 6 and Figure S13). The *E* isomer exhibited an intense absorption band centered at 302 nm, in good qualitative agreement with the experimental spectrum. This band arises from the extended and almost complete planar structure of the *E*-form, which enhances π conjugation between the phenyl ring and α, β-unsaturated amide fragment. In contrast, the *Z* isomer displayed a significantly weaker absorption band centered at 257 nm, related to a diminished π → π* transition of the cinnamoyl C=C, in excellent agreement with the experimental findings. These results demonstrate that only the *E* form can absorb light at the wavelength chosen for experimental irradiation and rationalize the significant decrease in the intensity of the experimentally observed band at 280 nm. It is worth noting that the band at 257 nm for the *Z* form can rationalize the slight blue shift in the main absorption band after irradiation, further confirming the experimental findings revealing the predominance of the *E* → *Z* photoisomerization pathway. However, this spectroscopic agreement should not be overinterpreted as a direct demonstration of the detailed excited-state mechanism.

In addition to *E* → *Z* photoisomerization, photodimerization may occur upon irradiation through reactions between (i) two *E* isomers, (ii) two *Z* isomers, and (iii) one *E* and one *Z* isomer. Each combination can react in either a head-to-head or head-to-tail mode. Consequently, many photodimerization scenarios are possible, and not all can be investigated computationally. To address this point, we examined three families of candidate photoproducts: (i) *E*/*E*, (ii) *Z*/*Z*, and (iii) *E*/*Z* isomeric pairs, each with two possible connectivities (head-to-head and head-to-tail). After geometry optimization, the lowest-energy structure in each family was retained as the representative for subsequent TD-DFT calculations (Figure 7, SI Section III and Figures S14–S17). Interestingly, while being structurally highly different, these photoproducts have very similar spectra, with pronounced blue shifts (*λ*_max_ between 180 and 190 nm) relative to the absorption bands of the *E* and *Z* isomers (Figure 7). The intense absorption peak associated with the photoproducts is clearly not observed in the experimental spectra, as indicated by the absence of significant changes in absorption intensity in the 200–220 nm range, consistent with the very low extent of photodimerization observed experimentally. It should be emphasized, however, that these calculations were not intended to describe the excited-state reaction pathway itself. No excited-state potential-energy surface, transition pathway, conical intersection, or non-adiabatic relaxation process was explicitly investigated in the present work. Therefore, the calculated spectra should be regarded as a spectroscopic support for the assignment of the experimentally observed species rather than as a direct mechanistic proof of the predominant *E* → *Z* photoisomerization pathway.

## 3. Materials and Methods

### 3.1. Materials

Methyl cinnamate (≥99%), diethanolamine (≥99%), dichloromethane (≥99.0%), and methanol-*d4* (deuteration degree minimum 99.8% for NMR spectroscopy) were purchased from Merck. Sodium methoxide (25 wt% in methanol) and NaCl salt (≥98%) were purchased from Sigma-Aldrich. Magnesium sulphate (anhydrous, 99.5%) was acquired from Alfa Aesar. Acetone (99.5%), N-heptane (99+–mixtures of isomers) and methanol (99.8%) were supplied by Chemlab. All reagents were used as received.

### 3.2. Experimental Procedures

#### 3.2.1. Synthesis of *E*-*N*,*N*-bis(2-Hydroxyethyl)cinnamamide (*E*-BHECA)

BHECA was synthesized and purified via the ammonolysis reaction between methyl cinnamate and an excess of diethanolamine catalyzed by sodium methoxide, as reported by Jin et al. [3] ^1^H NMR revealed a coupling constant (*J*) of 15.5 Hz for both H_d_ and H_e_ suggesting that this monomer exists predominantly in the *E*-configuration [18,20] (Figure S1). HPLC-ESI-MS analyses showed a single chromatographic peak at a retention time of 15.99 min and a base peak with *m*/*z* 236.0 [BHECA + H]^+^ (Figure S2) confirming the successful synthesis and purification of *E*-BHECA.

#### 3.2.2. Photoirradiation of E-BHECA in a Methanolic Solution

*E*-BHECA (30 mg) was dissolved in 3 mL of methanol-*d4* (10 mg/mL) in a quartz cuvette. Photoisomerization was induced by exposing the solution in the quartz tube to UV light at *λ* = 300–450 nm (irradiance of 60.649 mW/cm^2^) using a Dymax ECE 2000 series curing system equipped with a 400 W PN-38560 metal-halide bulb. Irradiation was performed at a distance of 10 cm from the light source under atmospheric conditions in the dark for definite irradiation times. Subsequently, ^1^H NMR was used to identify and quantify the nature of each species. The fractions of the species present in the mixtures (doublets at 7.18 and 6.22 ppm for *E* and *Z* respectively in Figure 1A, teal) were monitored via ^1^H NMR spectroscopy [11] using [Equations (S1) and (S2)].

#### 3.2.3. Photoirradiation of E-BHECA in Solid State

The photochemical reactions of *E*-BHECA in SS were induced by exposing 30 mg of powdered *E*-BHECA in a Petri dish to UV light at *λ* = 300–450 nm (irradiance of 60.649 mW/cm^2^) using a Dymax ECE 2000 series curing system equipped with a 400 W PN-38560 metal-halide bulb. Irradiation was performed at a distance of 10 cm from the light source under atmospheric conditions in the dark for definite irradiation times. Using ^1^H NMR spectroscopy, [11] the nature of each species in the mixture (doublets at 7.18, 6.22 and 3.97 ppm for *E*, *Z*, and dimer respectively in Figure 1A,B, black) was identified and quantified via [Equations (S3)–(S5)].

#### 3.2.4. Isolation and Characterization of Z-BHECA

To isolate and characterize the *Z*-form of BHECA, *E*-BHECA was subjected to photoisomerization under the conditions described above to yield an *E*/*Z*-BHECA mixture, which was separated by a preparative HPLC using formic acid in water and acetonitrile as the eluent. Fractions containing *Z*-BHECA were identified via ^1^H NMR spectroscopy, combined and concentrated under reduced pressure to afford *Z*-BHECA as a colorless oil. ^1^H NMR (CD_3_OD, 500 MHz): *δ* ppm 3.54–3.59 (dt, 4H, f’), 3.62–3.78 (dt, 4H, g’), 6.22 (d, *J* = 12.8 Hz, 1H, **e’**), 6.69 (d, *J* = 12.8 Hz, 1H, **d’**), 7.27–7.34 (m, 3H, a’, b’), 7.43 (d, 2H, c’). ESI-MS: *m*/*z* 236.0 [M + H]^+^.

### 3.3. Density Functional Theory (DFT) and TD-DFT Calculations

DFT calculations were performed using the Gaussian 09 (D01 revision) [44] suite to optimize the geometries of *E*-BHECA and its possible photoproducts. All geometry optimization calculations were conducted in the gas phase using the B3LYP functional [45,46,47] combined with the 6-31G** double-zeta basis set. To properly describe the dispersion interactions, Grimme’s empirical dispersion correction GD3BJ was applied [48]. In order to ensure local minima free of imaginary vibrational frequencies, a vibrational analysis of the geometrically optimized structures was conducted. For the DFT calculations, the following Gaussian keywords were used: B3LYP/6-31G** opt Freq EmpiricalDispersion=GD3BJ. Different starting points were considered for the geometry optimizations, and only the most stable structure was used for the simulations of the gas-phase absorption spectra. The gas-phase-optimized geometries of the most stable conformers were subsequently used for the simulations of the absorption spectra at the TD-DFT level, using the same functional and basis set. Solvent effects were included at the TD-DFT stage using the Polarizable Continuum Model (PCM), with methanol as the solvent [49]. For the TD-DFT calculations, the following Gaussian keywords were used: TD=(Singlets,NStates=50) B3LYP/6-31G** Pop=(NTO,Full) Density SCRF=(Solvent=Methanol,PCM) EmpiricalDispersion=GD3BJ. Despite all our computational efforts, due to their high flexibility, there is no guarantee that the lowest energy minimum cannot be even further stabilized. However, as explained in the Section 2, this will not have any impact on the conclusions related to the absorption spectra.

## 4. Conclusions

This study provides comprehensive insights into the intrinsic photoreactivity of *E*-BHECA through complementary experimental (^1^H NMR, HPLC-ESI-MS, UV–Vis and SC-XRD) and theoretical (TD-DFT) methods. At first glance, experimental investigations highlight the critical roles of two key parameters—the physical state of the reaction medium and irradiation time on the photochemical outcomes. Specifically, the photochemical impacts after six and ten hours of UV irradiation of *E*-BHECA in methanol and SS were investigated. The findings revealed selective *E* → *Z* photoisomerization in methanol achieving 38 ± 0.4% and 49 ± 0.4% conversion yield after six and ten hours of UV irradiation, respectively. In SS, *E* → *Z* photoisomerization remained dominant (31 ± 0.4% and 41 ± 0.7% after six and ten hours, respectively) but minor photodimerization also occurred, yielding four distinct dimers with unresolved stereochemistry at very low yields of 1 ± 0.2% and 2 ± 0.3% after six and ten hours respectively, together with minimal side reactions. This very low extent of photodimerization of *E*-BHECA in SS is related to the large distance of 5.025 Å between adjacent cinnamoyl C=C, as revealed by SC-XRD. Furthermore, the isolation and characterization of the major product, *Z*-BHECA, enabled unambiguous confirmation of the structural and spectroscopic assignments. At second glance, TD-DFT calculations further rationalized the predominance of *E* → *Z* photoisomerization pathway over minor photodimerization.

Future studies could investigate the influence of other key parameters, such as wavelength selectivity, on the outcomes and efficiency of the photochemical reactions. Supramolecular host–guest complex formation and non-covalent or covalent templates in solution or SS could also be explored to selectively enhance photoisomerization and photodimerization reactions of *E*-BHECA. Further work will also focus on determining the stereochemistry of the dimer products with enhanced photodimerization extent.

This work not only advances our knowledge of *E*-BHECA’s photochemical reactivity under defined conditions but also paves the way for future potential applications in material science, organic synthesis and pharmaceuticals.

## Figures and Tables

**Scheme 1:**
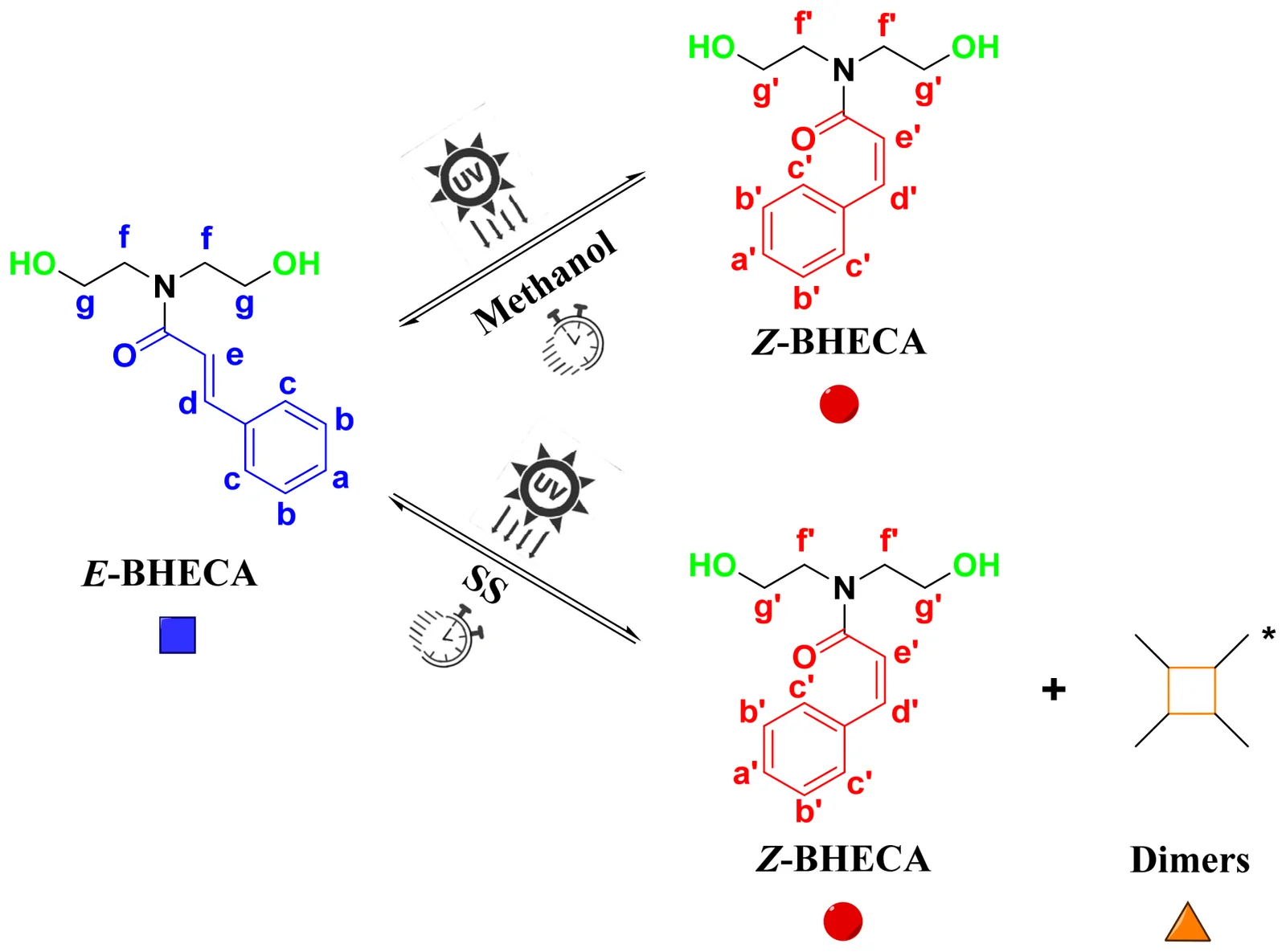
Photochemical reactions of *E*-BHECA upon UV irradiation in a methanolic solution and in the SS at various irradiation times (* for clarity, only a generic form of dimers was illustrated).

**Figure 1:**
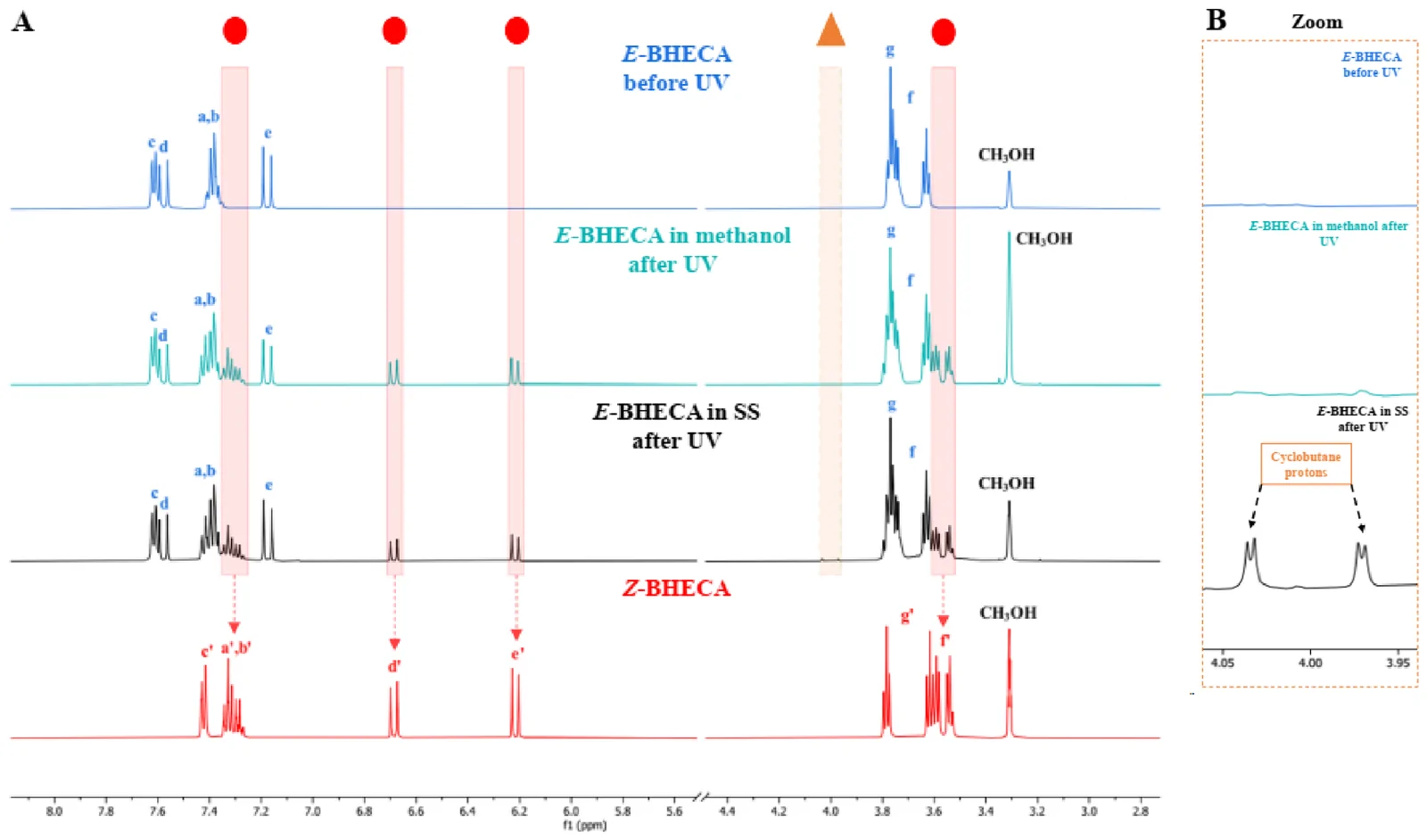
(**A**) ^1^H NMR (500 MHz, CD_3_OD) spectra of *E*-BHECA before UV irradiation (blue), after six hours of UV irradiation in methanol (teal), after six hours of UV irradiation in SS (black) and isolated *Z*-BHECA (red), with colored symbols indicating the nature of each species namely, *Z*-BHECA (red circle) and dimer (orange triangle); (**B**) zoom between 3.95 ppm and 4.05 ppm of Figure 1 (**A**), blue, teal and black.

**Figure 2:**
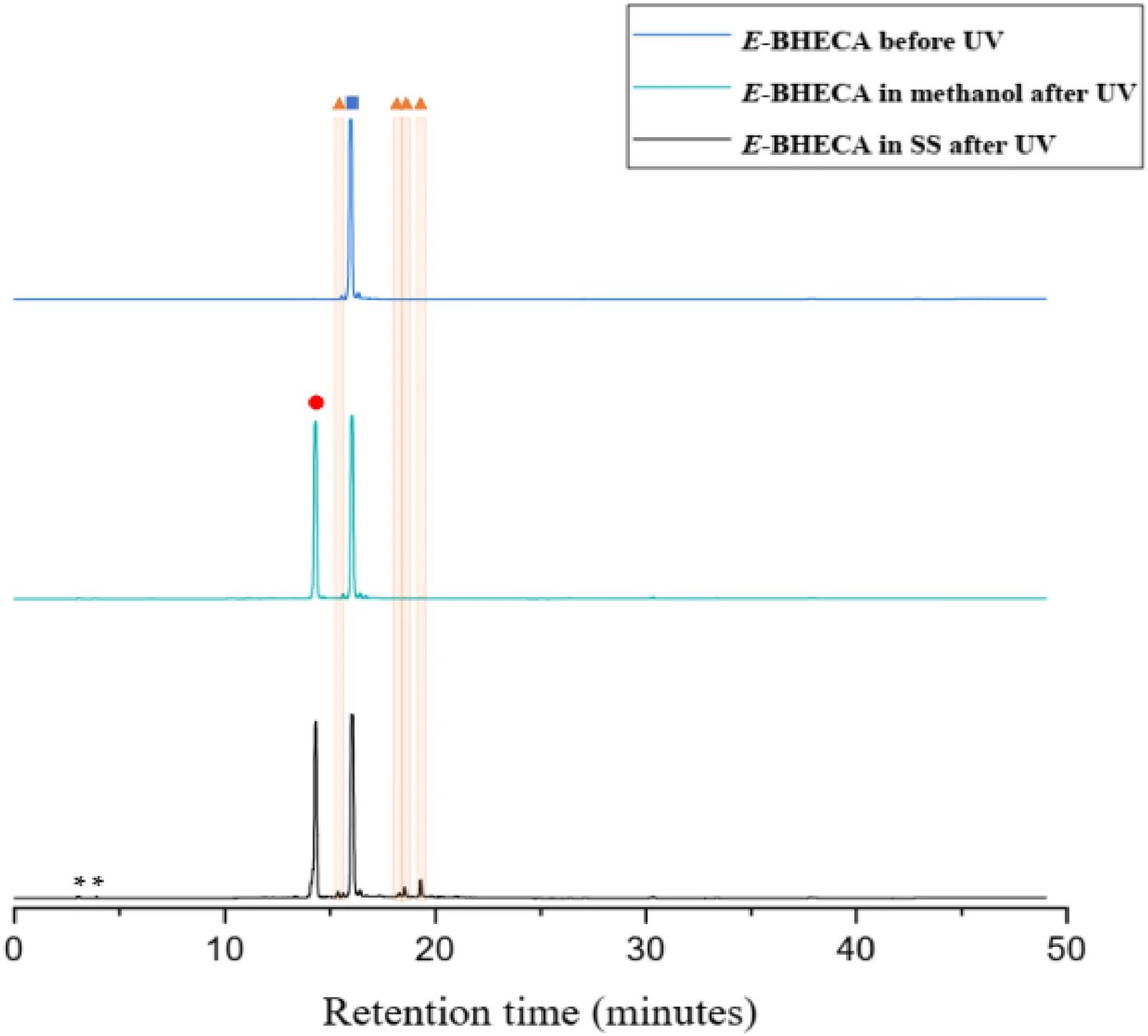
HPLC chromatograms of *E*-BHECA before UV irradiation (blue), after six hours of UV irradiation in methanol (teal) and after six hours of UV irradiation in SS (black). The colored symbols, blue square, red circle, orange triangles, and asterisk, represent *E*-BHECA, *Z*-BHECA, dimer products, and side reaction products respectively.

**Figure 3:**
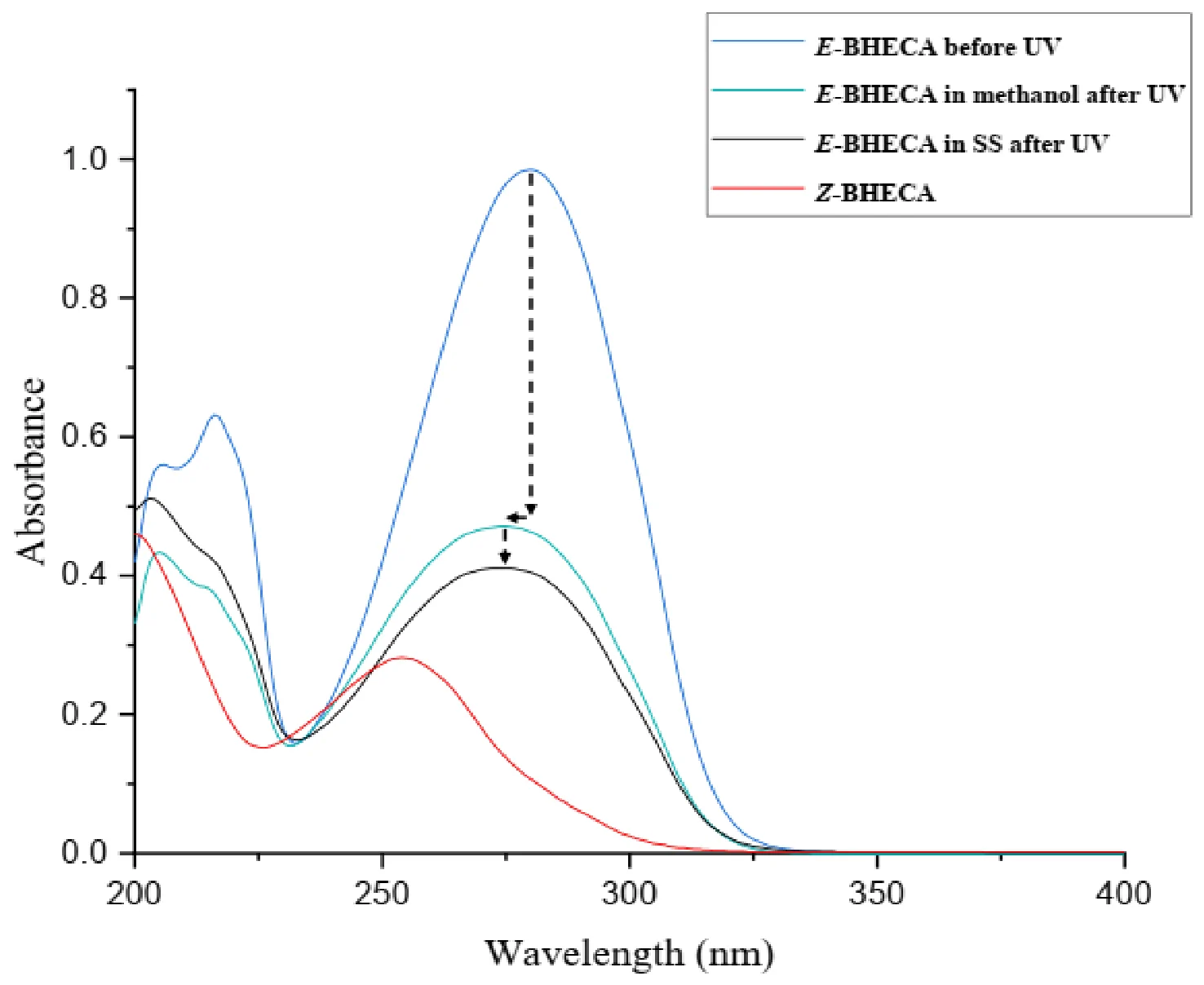
UV–Vis spectra of *E*-BHECA before UV irradiation (blue), after six hours of UV irradiation in methanol (teal), after six hours of UV irradiation in solid state (black) and isolated *Z*-BHECA (red). Dashed vertical and horizontal arrows represent a decrease in absorbance and blue shift respectively.

**Figure 4:**
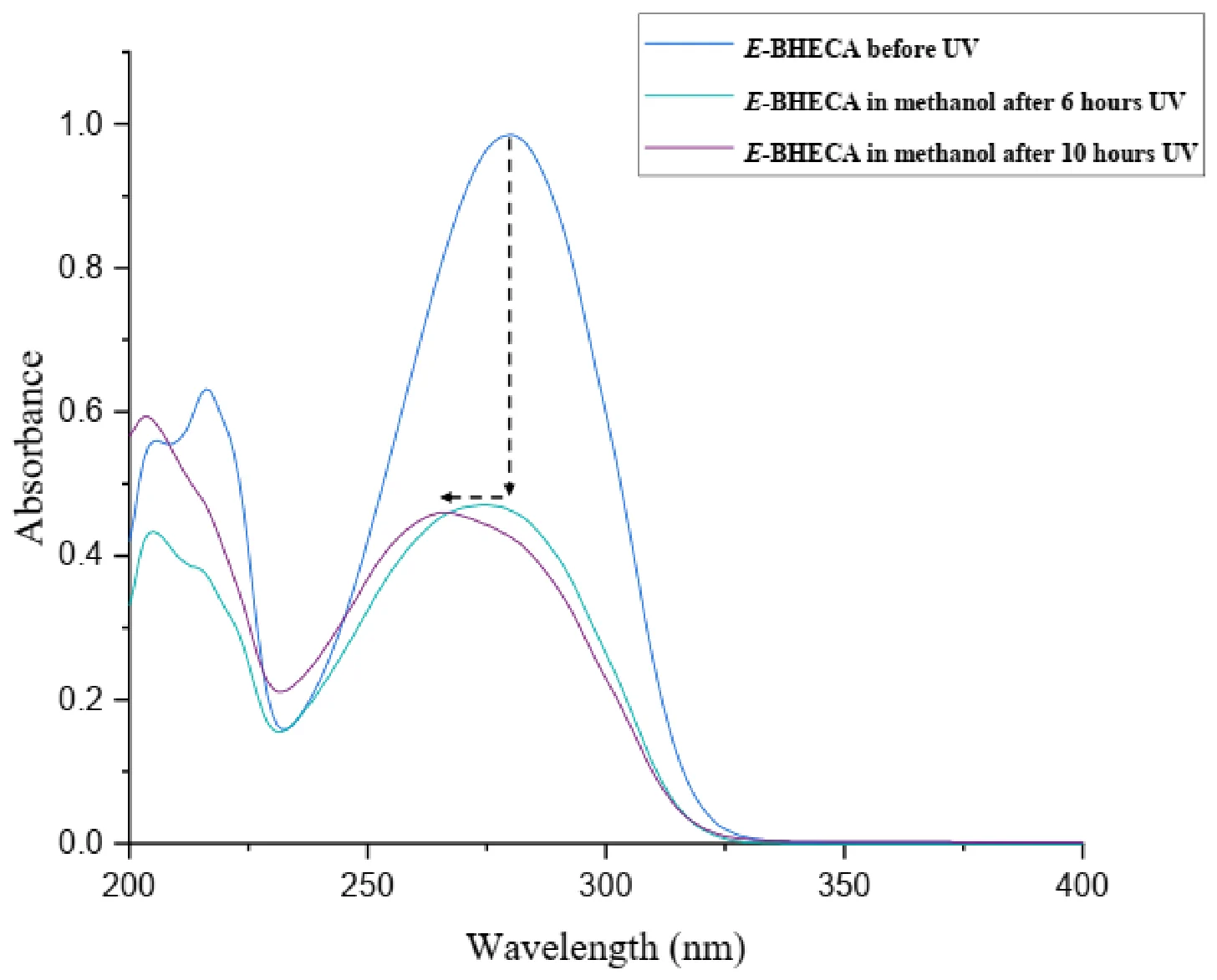
UV–Vis spectra of *E*-BHECA before UV irradiation (blue), after six hours of UV irradiation (teal) and after ten hours of UV irradiation (purple) in methanol. Dashed vertical and horizontal arrows represent a decrease in absorbance and blue shift respectively.

**Figure 5:**
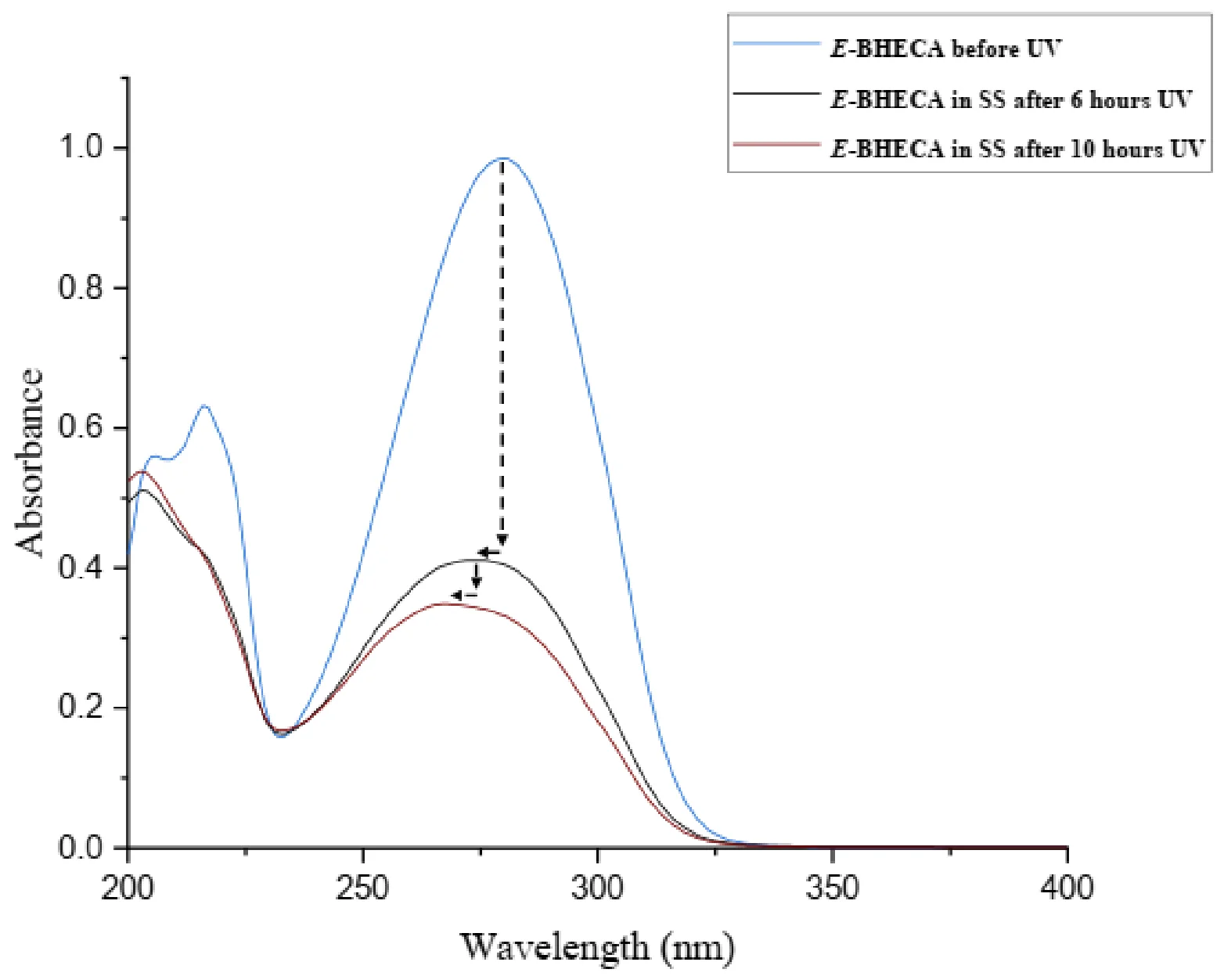
UV–Vis spectra of *E*-BHECA before UV irradiation (blue), after six hours of UV irradiation (black) and after ten hours of UV irradiation (brown) in SS. Dashed vertical and horizontal arrows represent a decrease in absorbance and blue shift respectively.

**Figure 6:**
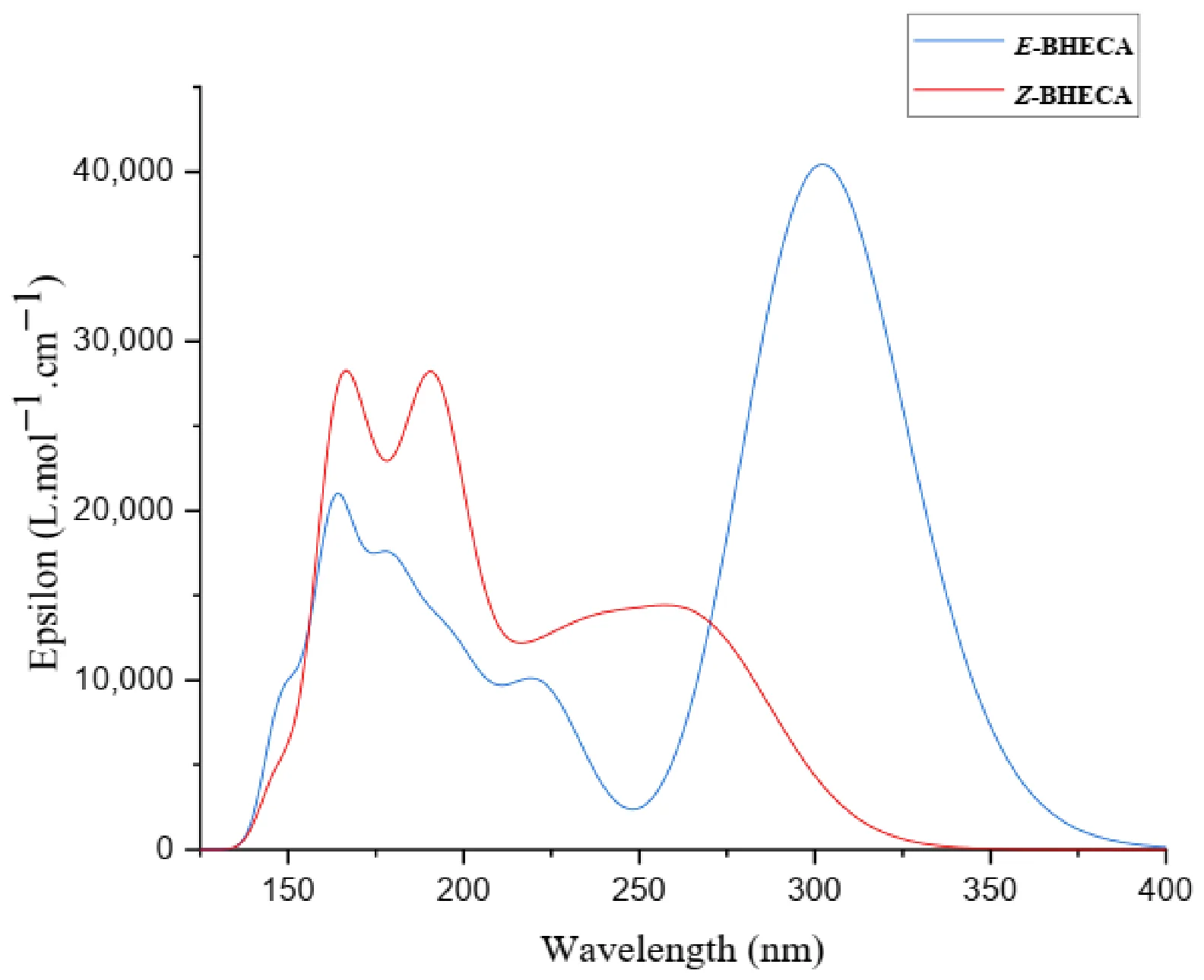
Theoretical UV–Vis spectra of *E*-BHECA (blue) and *Z*-BHECA (red), employing the TD-DFT formalism with the B3LYP functional, a 6-31G** basis set, GD3BJ as Grimme’s empirical dispersion correction and the Polarizable Continuum Model (PCM) with methanol as the solvent.

**Figure 7:**
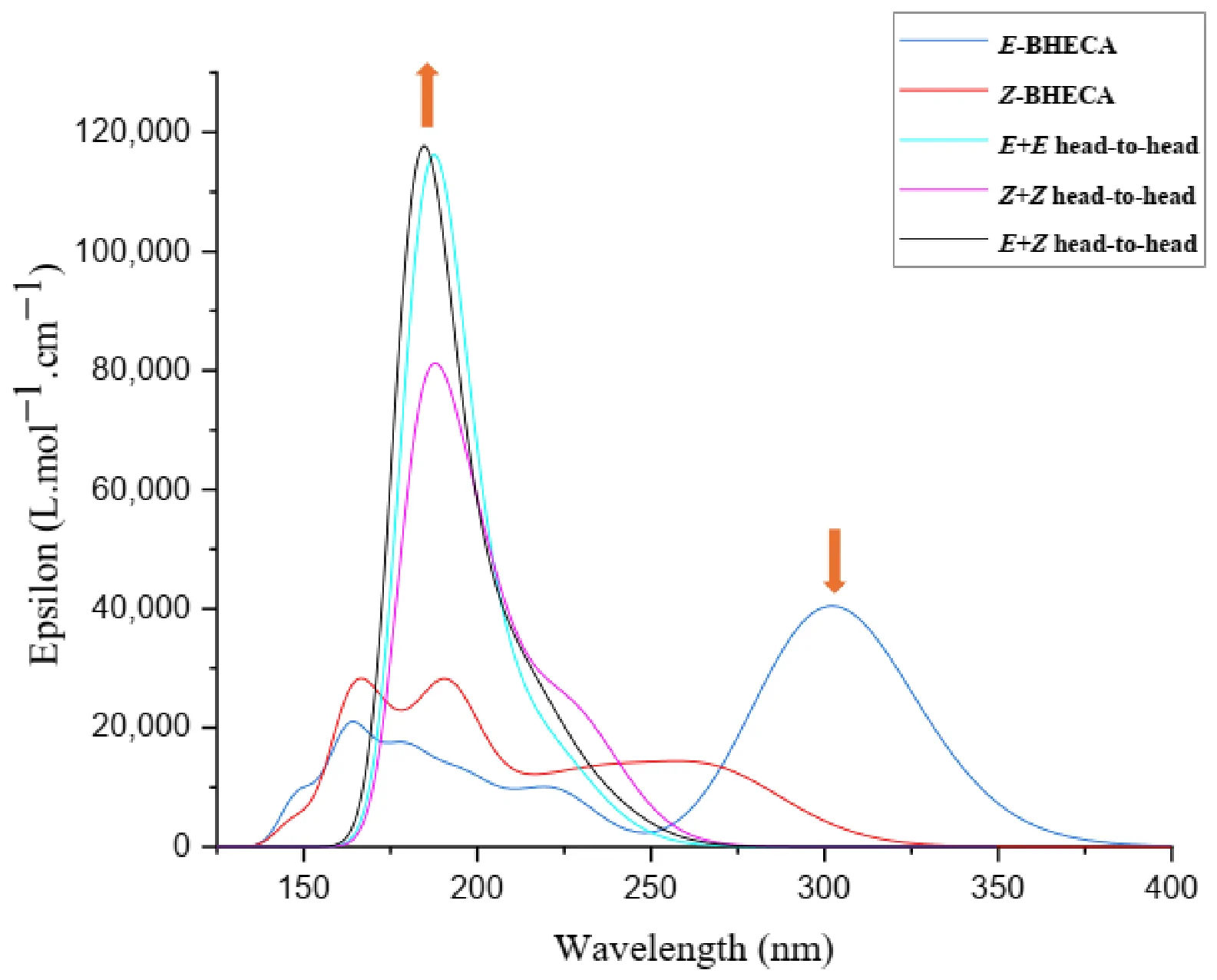
Theoretical UV–Vis spectra of *E*-BHECA (blue), *Z*-BHECA (red), head-to-head mode photodimerization product between two *E* isomers (cyan), head-to-head mode photodimerization product formed by two *Z* isomers (magenta) and head-to-head product between one *E* and one *Z* isomer (black), employing the TD-DFT methodology with the B3LYP functional, a 6-31G** basis set, GD3BJ Grimme’s empirical dispersion correction and the Polarizable Continuum Model (PCM) with methanol as the solvent. Arrows show intense absorption peaks associated with photodimers relative to the monomers.

**Table 1:** Mole fractions (%) of *E*-BHECA, *Z*-BHECA, and dimer before UV and after six and ten hours of UV irradiation in methanol and SS.

Entry	Physical State of the Medium	Irradiation Time (Hours)	^1^H NMR ^b,c^		
			*E* Fraction (%)	*Z* Fraction (%)	Dimer Fraction (%)
1	*E*-BHECA before UV	0	100	0	0
2	Methanol ^a^	6	62 ± 0.4	38 ± 0.4	0
		10	51 ± 0.4	49 ± 0.4	0
3	SS ^a^	6	68 ± 0.6	31 ± 0.4	1 ± 0.2
		10	57 ± 0.7	41 ± 0.7	2 ± 0.3

^a^ Samples exposed to 300–450 nm UV light. ^b^ Values are presented as mean ± standard deviation, *n* = 3. ^c^ For clarity, the reported percentages of the major identifiable photoproducts (*E*, *Z*, and dimer species) determined via ^1^H NMR were normalized to 100%, thereby excluding additional minor unresolved side products.

**Table 2:** HPLC-ESI-MS results summarizing the nature of species, retention times (T_r_), assigned ions and *m*/*z* values of *E*-BHECA before UV and after six and ten hours of UV irradiation in methanol and SS.

Entry	Physical State of the Medium	Irradiation Time (Hours)	Species	HPLC	ESI-MS	
				Retention Time-T_r_ (Minutes)	Ion	*m*/*z* ^b^
1	*E*-BHECA before UV	0	*E*	15.99	[BHECA + H]^+^	236.0
2	Methanol ^a^	6	*Z*	14.29	[BHECA + H]^+^	236.0
			*E*	16.03	[BHECA + H]^+^	236.0
		10	*Z*	14.30	[BHECA + H]^+^	236.0
			*E*	16.05	[BHECA + H]^+^	236.0
3	SS ^a^	6	*Z*	14.30	[BHECA + H]^+^	236.0
			*D*_1_	15.37	[(BHECA)_2_ + H]^+^	471.1
			*E*	16.01	[BHECA + H]^+^	236.0
			*D*_2_	18.30	[(BHECA)_2_ + H]^+^	471.1
			*D*_3_	18.54	[(BHECA)_2_ + H]^+^	471.1 *
			*D*_4_	19.30	[(BHECA)_2_ + H]^+^	471.1
		10	*Z*	14.30	[BHECA + H]^+^	236.0
			*D*_1_	15.36	[(BHECA)_2_ + H]^+^	471.1
			*E*	16.04	[BHECA + H]^+^	236.0
			*D*_2_	18.29	[(BHECA)_2_ + H]^+^	471.1
			*D*_3_	18.53	[(BHECA)_2_ + H]^+^	471.1 *
			*D*_4_	19.29	[(BHECA)_2_ + H]^+^	471.1

^a^ Samples exposed to 300–450 nm UV light. ^b^ *m*/*z* values of the base peaks in the mass spectra (refer to Supplementary Materials Figures S4, S5, S7 and S11 for the mass spectra). * *D*_3_ has a molecular ion peak and a base peak with *m*/*z* of 471.1 and 366.0 respectively. The base peak of *D*_3_ with *m*/*z* of 366.0 results from the cleavage of an amide bond (N-CO) in the dimer during ionization.

## Data Availability

The data presented in this study are available on request from the corresponding author.

## Supplementary Materials

The following supporting information can be downloaded at: https://www.mdpi.com/article/10.3390/molecules31193434/s1, Instrumentation, ^1^H NMR-based quantification equations, ground-state thermodynamic stability of monomers and possible photodimers, supplementary figures, table and list of the cartesian coordinates of all optimized structures are found in the Supplementary Materials (SM). Figure S1. ^1^H NMR (500 MHz, CD_3_OD) spectrum of *E*-BHECA; Figure S2. (A) HPLC chromatogram and (B) ESI-MS spectrum of *E*-BHECA; Figure S3. (A) ^1^H NMR (500 MHz, CD_3_OD) spectra of *E*-BHECA upon six hours of UV irradiation, and after two weeks at room temperature in the dark in methanol (A) and (B) SS; Figure S4. Extracted ESI-MS spectra of *Z*-BHECA and *E*-BHECA obtained after six hours of UV irradiation in methanol; Figure S5. Extracted ESI-MS spectra of *Z*-BHECA, *D*_1_, *E*-BHECA, *D*_2_, *D*_3_ and *D*_4_ after six hours of UV irradiation in solid state; Figure S6. HPLC chromatograms of *E*-BHECA before UV irradiation (blue), after six hours of UV irradiation (teal) and after ten hours of UV irradiation (purple) in methanol. Colored symbols; blue square and red circle represent *E*-BHECA and *Z*-BHECA respectively; Figure S7. Extracted ESI-MS spectra of *Z*-BHECA and *E*-BHECA obtained after ten hours of UV irradiation in methanol; Table S1. Crystal data and structure refinement details for *E*-BHECA; Figure S8. Crystal structure of *E*-BHECA including multiple viewing angles obtained from SC-XRD analysis; Figure S9. P-XRD spectra of solid *E*-BHECA (blue) and SC-*E*-BHECA (red); Figure S10. HPLC chromatograms of *E*-BHECA before UV irradiation (blue), after six hours of UV irradiation (black) and after ten hours of UV irradiation (brown) in SS. Colored symbols; blue square, red circle, orange triangles, and asterisk represent *E*-BHECA, *Z*-BHECA, dimer products, and side reactions products respectively; Figure S11. Extracted ESI-MS spectra of *Z*-BHECA, *D*_1_, *E*-BHECA, *D*_2_, *D*_3_ and *D*_4_ after ten hours of UV irradiation in solid state; Figure S12. *E* → *Z* photoisomerization kinetics upon UV irradiation of *E*-BHECA in methanol; Figure S13. Representation of the energies and contour plots of the frontier molecular orbitals calculated at the DFT/B3LYP/6-31G**/GD3BJ level, including solvent effects through the Polarizable Continuum Model (PCM) with methanol as the solvent, for *Z*-BHECA (left) and *E*-BHECA (right); Figure S14. Representation of photodimer 1 which consists of a [2π + 2π] photocycloaddition reaction product between two *E* isomers in a head-to-head combination mode. Light blue line is used to indicate the hydrogen bonds; Figure S15. Representation of photodimer 2 which consists of a [2π + 2π] photocycloaddition reaction product between two *Z* isomers in a head-to-head combination mode. Green lines are used to indicate the hydrogen bonds as well as C-H-π interactions; Figure S16. Representation of photodimer 3 which consists of a [2π + 2π] photocycloaddition reaction product between one *E* and one *Z* isomers in a head-to-head combination mode. Light blue line is used to indicate the hydrogen bonds; Figure S17. Representation of the energies and contour plots of the frontier molecular orbitals calculated at the DFT/B3LYP/6-31G**/GD3BJ level, including solvent effects through the Polarizable Continuum Model (PCM) with methanol as the solvent, for the of three photoproducts considered in this work. Additional references cited within the SM [50,51,52].
