# Supplementary material for: Photoreactivity of E-N,N-bis(2-hydroxyethyl)cinnamamide in Methanol and Solid State: A Complementary Experimental and Theoretical Investigation
Source: Molecules. 2026 Sep 27;31(19):3434. doi: 10.3390/molecules31193434 (PMC13665741; doi:10.3390/molecules31193434)
Supplement: Supplementary file 1 [file molecules-31-03434-s001.zip › molecules-4485515-supplementary.pdf]

Supplementary Materials

to

Photoreactivity of *E*-*N*,*N*-bis(2-hydroxyethyl)cinnamamide in methanol and solid state: A complementary experimental and theoretical investigation

Jacques Kevin Wandji [1,2,*], Sarajit Naskar [3], Vincent Lemaur [3], Claudio Palmieri [4], Amandine Nachtergael [4], Koen Robeyns [5], Jérôme Cornil [3], Rosica Mincheva [1] and Jean-Marie Raquez [1,2]

[1] Laboratory of Polymeric and Composite Materials (LPCM), University of Mons, Place du Parc 20, 7000 Mons, Belgium.

[2] WEL Research Institute, Avenue Pasteur 6, 1300 Wavre, Belgium.

[3] Laboratory for Chemistry of Novel Materials, University of Mons, Place du Parc 20, 7000 Mons, Belgium.

[4] Unit of Therapeutic Chemistry and Pharmacognosy, University of Mons, Place du Parc 20, 7000 Mons, Belgium.

[5] Molecular Chemistry, Materials and Catalysis (MOST), Institute of Condensed Matter and Nanosciences (IMCN), Université Catholique de Louvain,1348 Louvain-la-Neuve, Belgium.

* Correspondence: jacques.wandji@umons.ac.be

Table of contents

I. Instrumentation ……………………………………………………………………….3

II. ^{1}H NMR-based quantification equations …………………………………………..4

III. Ground-state thermodynamic stability of monomers and possible photodimers…..5

IV. Supplementary figures and table..……………………………………….................5

V. List of the cartesian coordinates of all optimized structures………………………17

VI. References ……………………………………………………………………..22

I. Instrumentation

Flash chromatography was used for the purification of *E*-BHECA. The instrument model used was a Biotage® Isolera TM Spectra One and Four and the normal phase silica column used was a Biotage® SNAP cartridge KP-SIL 120g, equipped with a UV-Visible detector (254 and 280 nm). The pressure in the machine was maintained between 0 and 5 bars.

The ^{1}H NMR spectra were recorded on a Bruker Avance II-500 MHz at room temperature in methanol-*d4* (CD_3OD). The number of scans was 256. The chemical shift values (δ) were measured in parts per million (ppm) on the scale with tetramethylsilane (TMS) as an internal reference. Signal multiplicities were reported as: doublet (d), doublet of triplets (dt) and multiplet (m). Coupling constants (*J*) are reported in Hertz (Hz).

LC-MS measurements were performed on an HPLC Agilent (model 1260 Infinity II, Agilent Technologies, Santa Clara, CA, USA) system equipped with a DAD detector. Separation was performed using a Luna® 5 µm C18(2) 100 Å, 250 × 4.6 mm column (Phenomenex, Torrance, California, USA) maintained at 25 °C. The injection volume was of 5 µL and the wavelength was set at 210 nm specifically for dimer detection. The separation of compounds was achieved at a flow rate of 1.0 mL/min using 1.0 % v/v formic acid in water (solvent A) and acetonitrile (solvent B) as mobile phases with an elution gradient as follows: 0-2 min, 5.0 % B; 2-47 min, 100.0 % B; 47-49 min, 5.0 % B. The flow of components eluting from the chromatographic column was split in a 1:10 ratio where 10 % of the eluent was directly injected into the mass spectrometer (Single quadrupole analyzer, model Expression^{L} CMS, Advion, Ithaca, NY, USA) through an electrospray ionization pressure chemical ionization interface using a post-column passive splitter. Mass spectrometric detection was performed in positive ESI mode and the temperature levels of ESI interface, desolvation line, and heat block were 250 °C and the flows of nebulizing gas (N_2), heating gas (air) were set at 5 L/min. Full scan mass spectra were registered in positive mode between 200 and 550 *m*/*z* (2.5 kV source voltage and collision energy from -1.2-0.435 eV) with a scan speed of 1000 *m*/*z*/sec . MassExpress.5.1.0.2 software was used to manage the HPLC-ESI-MS system. The final identification of each species was done after integrated analyses of retention times and mass spectra.

Isolation of *Z*-BHECA was performed on a BUCHI® preparative HPLC (Pure C-850 Flashprep, BÜCHI Labortechnik AG, Flawil, Switzerland) system equipped with a DAD detector. Isolation was performed using a Robusta 5 µm C18, 150 × 30 mm column (SepaChrom, Rho, Italy) maintained at 25 °C. The injection volume was 500 µL (133.3 mg/mL) and the

wavelength was set at 250 nm specifically for the detection of *Z*-BHECA. The purification of *Z*-BHECA was achieved at a flow rate of 40.0 mL/min using 1.0 % v/v formic acid in water (solvent A) and acetonitrile (solvent B) as mobile phases with an elution gradient as follows: 0-1 min, 5.0 % B; 1-17 min, 60.0 % B; 17-23 min, 100.0 % B; 23-24 min, 5.0 % B.

The UV-Visible spectra were recorded on a Shimadzu ® UV-2600 UV-Vis spectrophotometer in a 3 mL quartz cuvette with a light path of 10 mm over a wavelength range of 200-400 nm with a data interval of 1 nm. It was used to monitor the intensity changes and shift of the $\pi \rightarrow \pi^*$ transition of the cinnamoyl C=C of *E*-BHECA centered at 280 nm. The blank reference was methanol, and the concentrations of the solutions were 3.20×10^{-5} M.

Single crystal diffraction data were recorded on a MAR345 image plate detector under ambient conditions using Mo Kα radiation (λ = 0.71073 Å), generated by an Incoatec IµS microfocus source (Montel mirrors). Data integration and reduction were performed by CrysAlis^{PRO},[1] and the implemented absorption correction was applied. The structure solution was performed by dual-space direct methods in SHELXT[2] and the structure was further refined against F^2 using SHELXL2019/3.[3] Non-hydrogen atoms were refined anisotropically; H_{CH} atoms were added in calculated positions, H_{OH} atoms were located from the Fourier map, and the distance was allowed to refine, hydrogen atoms were refined with isotropic displacement factors set to 1.2 Ueq of the parent atoms (1.5 Ueq for OH hydrogens). Single crystals of *E*-BHECA suitable for XRD measurement were grown by vapor diffusion of pentane into a chloroform solution of *E*-BHECA. CCDC 2495424 includes the supplementary crystallographic data and can be downloaded free of charge from The Cambridge Crystallographic Data Centre (CCDC) via www.ccdc.cam.ac.uk/structures. The crystallographic data for *E*-BHECA are reported in **Table S1**.

II. ¹H NMR-based quantification equations

$$E \text{ fraction } (f_E)\ (\%) = \frac{\int E\text{–}vinylene}{\int E\text{–}vinylene + \int Z\text{–}vinylene}\ x\ 100\% \qquad (S1)$$

$$Z \text{ fraction } (f_Z)\ (\%) = \frac{\int Z\text{–}vinylene}{\int E\text{–}vinylene + \int Z\text{–}vinylene}\ x\ 100\% \qquad (S2)$$

$$E \text{ fraction } (f_E)\ (\%) = \frac{\int E\text{–}vinylene}{\int E\text{–}vinylene + \int Z\text{–}vinylene + \frac{\int cyclobutane}{2}}\ x\ 100\% \qquad (S3)$$

$$Z \text{ fraction } (f_Z)\ (\%) = \frac{\int Z\text{–}vinylene}{\int E\text{–}vinylene + \int Z\text{–}vinylene + \frac{\int cyclobutane}{2}}\ x\ 100\% \qquad (S4)$$

$$\text{Dimer fraction } (f_D)\ (\%) = \frac{\frac{\int cyclobutane}{2}}{\int E\text{−}vinylene + \int Z\text{−}vinylene + \frac{\int cyclobutane}{2}}\ x\ 100\% \qquad (S5)$$

Where E and Z correspond to trans and cis configurations respectively.

III. Ground-state thermodynamic stability of monomers and possible photodimers

The relative ground-state thermodynamic stability of *E*-BHECA, *Z*-BHECA, and the possible head-to-head photodimers was assessed from their calculated Gibbs free energies.

For the *E/Z* isomerization, the Gibbs free-energy difference was calculated as:

$$\Delta G_{E\rightarrow Z} = G(Z) - G(E)$$

The calculations indicate that E-BHECA is more stable than Z-BHECA by approximately **14.3 kcal mol^{-1}** in the ground state.

For the photodimers, no pre-associated non-covalent dimer complex was considered. Therefore, the stabilization free energy was defined relative to the corresponding isolated molecular fragments according to:

$$\Delta G_{\mathrm{stab}} = G(\mathrm{photoproduct}) - \sum G(\mathrm{isolated\ fragments})$$

The calculated stabilization free energies are summarized below:

**System**	**ΔGstab (kcal mol^{-1})**
2 *Z*-BHECA head-to-head photoproduct	−88.36
2 *E*-BHECA head-to-head photoproduct	−62.49
E+*Z*-BHECA head-to-head photoproduct	−78.45

All three possible photodimers are thermodynamically stabilized relative to the corresponding isolated fragments. Among the structures considered, the *Z/Z* head-to-head photoproduct exhibits the largest stabilization.

These ground-state thermodynamic data are intended to provide an energetic comparison between the different species and should not be interpreted as predicting the photochemical product distribution, which also depends on excited-state reaction pathways and kinetic factors.

IV. Supplementary figures and table

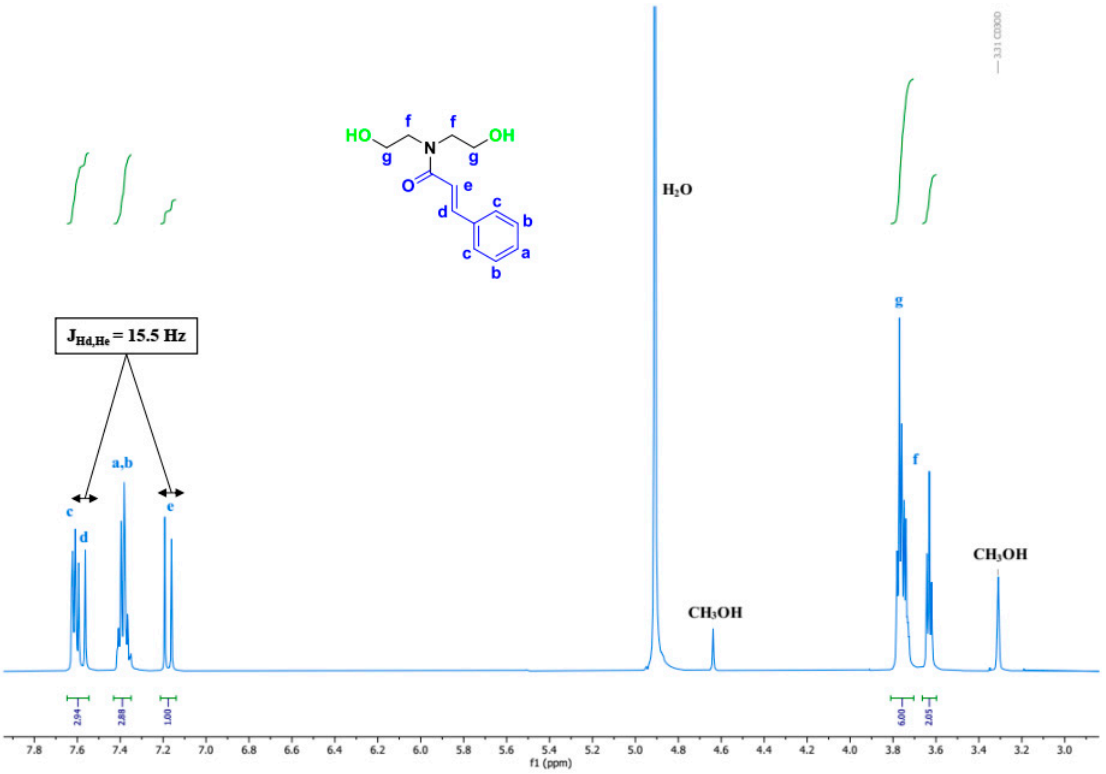

Figure S1. ^{1}H NMR (500 MHz, CD_3OD) spectrum of *E*-BHECA.

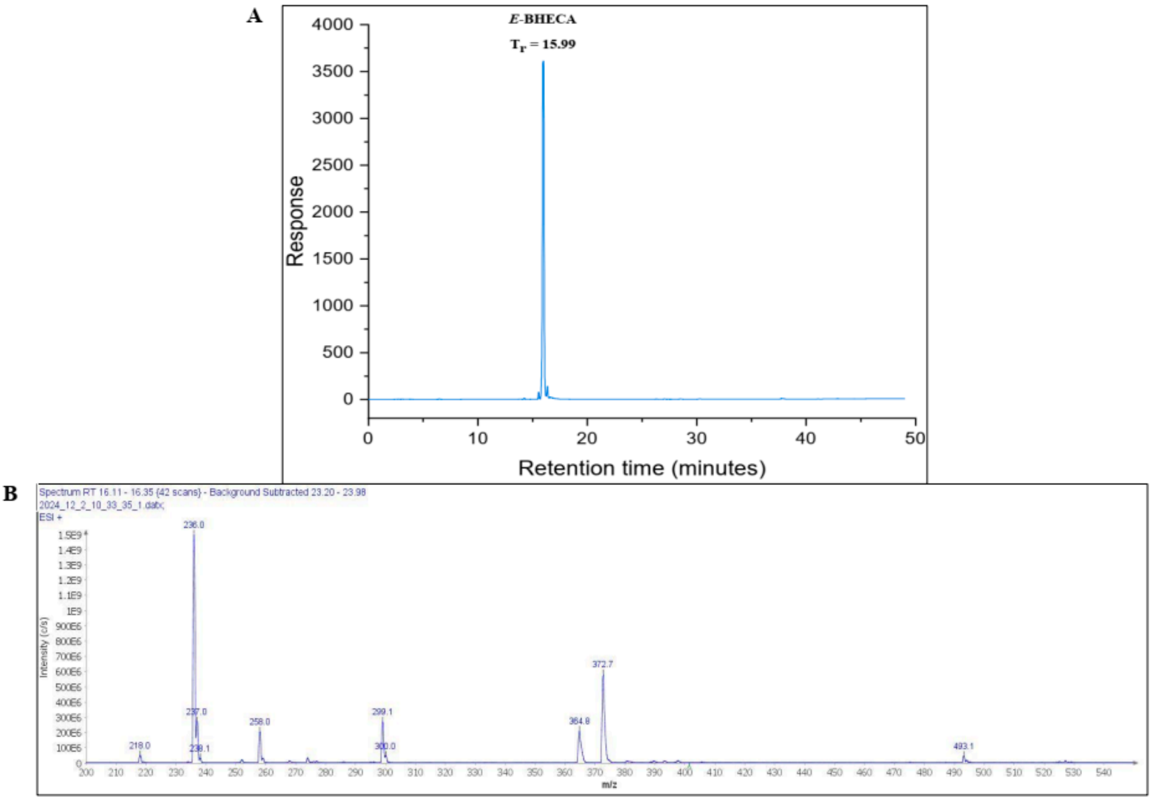

Figure S2. (A) HPLC chromatogram and (B) ESI-MS spectrum of *E*-BHECA.

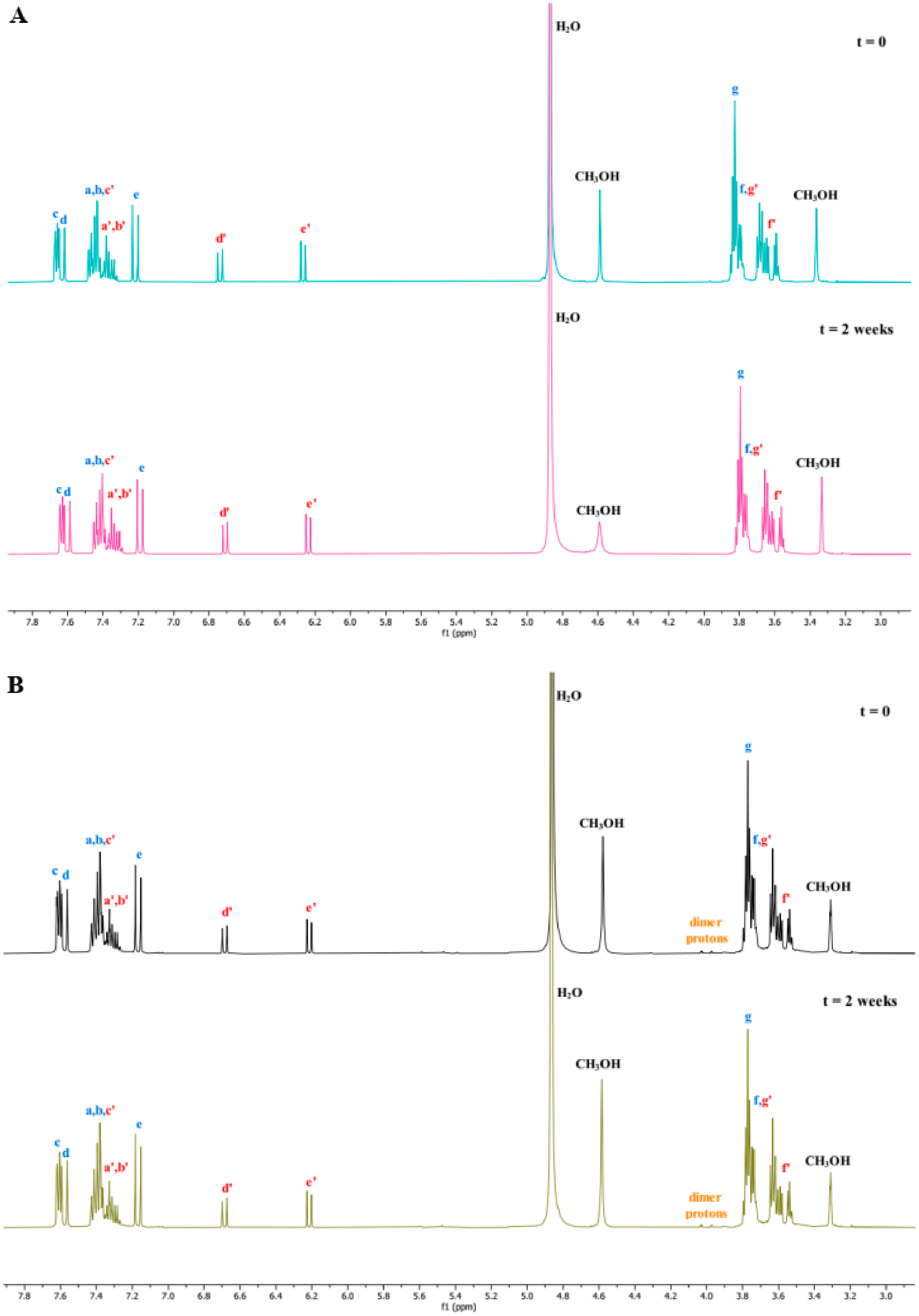

Figure S3. (A) ^{1}H NMR (500 MHz, CD_3OD) spectra of *E*-BHECA upon six hours of UV irradiation, and after two weeks at room temperature in the dark in methanol (A) and (B) SS.

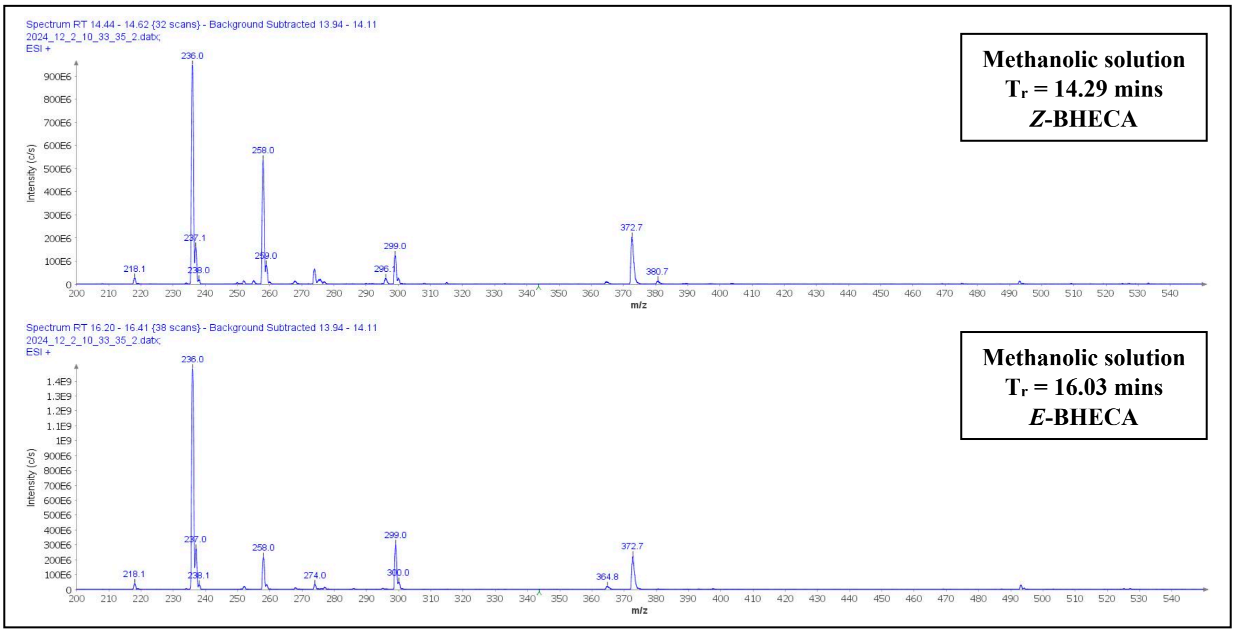

Figure S4. Extracted ESI-MS spectra of *Z*-BHECA and *E*-BHECA obtained after six hours of UV irradiation in methanol.

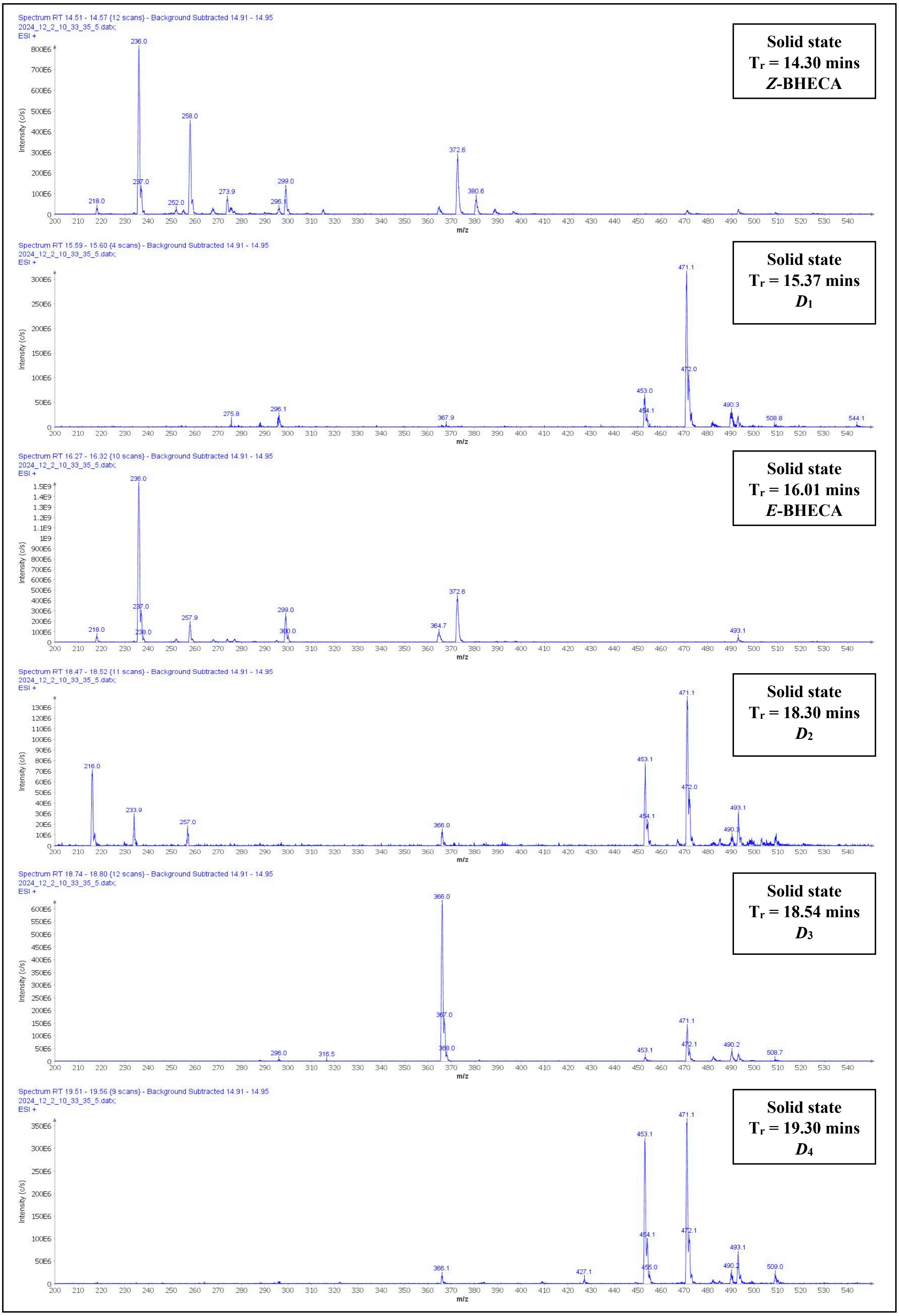
Spectrum RT 14.51 - 14.57 {12 scans} - Background Subtracted 14.91 - 14.95
2024_12_2_10_33_35_5.datx;
ESI +
Solid state
Tr = 14.30 mins
Z-BHECA
236.0
258.0
372.6
237.0
380.6
218.0
252.0
273.9
296.1
299.0
Intensity (c/s)
m/z
Spectrum RT 15.59 - 15.60 {4 scans} - Background Subtracted 14.91 - 14.95
2024_12_2_10_33_35_5.datx;
ESI +
Solid state
Tr = 15.37 mins
D1
471.1
472.0
453.0
454.1
490.3
508.8
544.1
275.8
296.1
367.9
Spectrum RT 16.27 - 16.32 {10 scans} - Background Subtracted 14.91 - 14.95
2024_12_2_10_33_35_5.datx;
ESI +
Solid state
Tr = 16.01 mins
E-BHECA
236.0
237.0
238.0
218.0
257.9
299.0
300.0
364.7
372.6
493.1
Spectrum RT 18.47 - 18.52 {11 scans} - Background Subtracted 14.91 - 14.95
2024_12_2_10_33_35_5.datx;
ESI +
Solid state
Tr = 18.30 mins
D2
471.1
453.1
472.0
454.1
493.1
490.3
216.0
233.9
257.0
366.0
Spectrum RT 18.74 - 18.80 {12 scans} - Background Subtracted 14.91 - 14.95
2024_12_2_10_33_35_5.datx;
ESI +
Solid state
Tr = 18.54 mins
D3
366.0
367.0
368.0
296.0
316.5
453.1
471.1
472.1
490.2
508.7
Spectrum RT 19.51 - 19.56 {9 scans} - Background Subtracted 14.91 - 14.95
2024_12_2_10_33_35_5.datx;
ESI +
Solid state
Tr = 19.30 mins
D4
471.1
453.1
454.1
472.1
493.1
490.2
509.0
455.0
427.1
366.1

Figure S5. Extracted ESI-MS spectra of *Z*-BHECA, D_1, *E*-BHECA, D_2, D_3 and D_4 after six hours of UV irradiation in solid state.

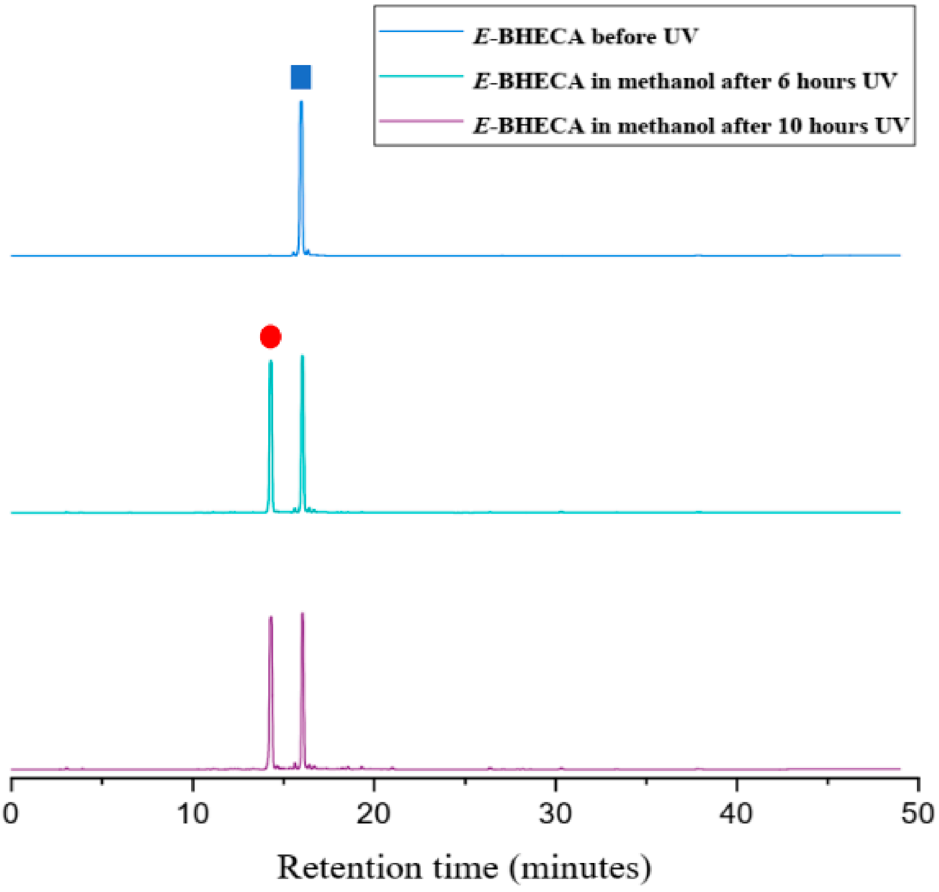

Figure S6. HPLC chromatograms of *E*-BHECA before UV irradiation (blue), after six hours of UV irradiation (teal) and after ten hours of UV irradiation (purple) in methanol. Colored symbols; blue square and red circle represent *E*-BHECA and *Z*-BHECA respectively.

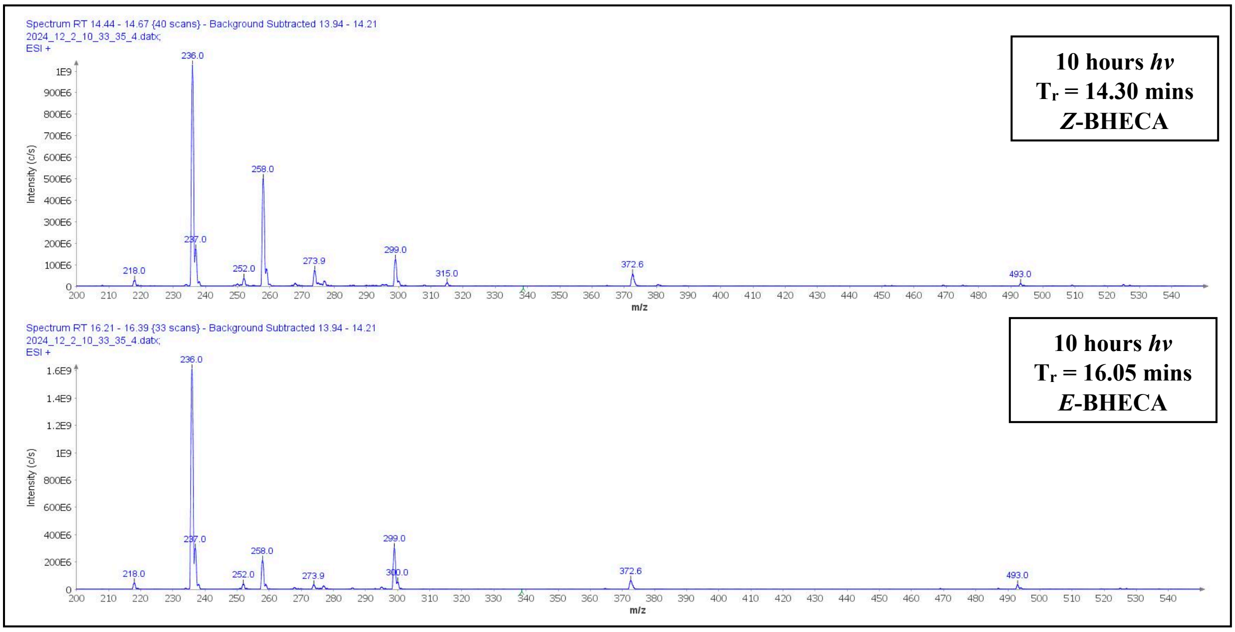

Figure S7. Extracted ESI-MS spectra of *Z*-BHECA and *E*-BHECA obtained after ten hours of UV irradiation in methanol.

Table S1. Crystal data and structure refinement details for *E*-BHECA.

Empirical formula	$C_{13}H_{17}NO_3$
Formula weight	235.27
Temperature (K)	297(2)
Wavelength (Å)	0.71073
Crystal system	Orthorhombic
Space group	$Pna2_1$
Unit cell dimensions (Å,°)	a = 28.707(2)
	b = 5.0246(4)
	c = 8.7675(6)
	a= 90
	b= 90
	g = 90
Volume (Å^3)	1264.61(17)
Z	4
Density (calculated) (g/cm^3)	1.236
Absorption coefficient (mm^{-1})	0.088
F(000)	504
Crystal size (mm^3)	0.50x 0.50 x 0.09
Theta range for data collection (°)	4.117 to 26.376
Reflections collected	8249
Independent reflections	2544 [R(int) = 0.0283]
Completeness to q = 25.242° (%)	99.3
Absorption correction	Semi-empirical from equivalents
Max. and min. transmission	1.00000 and 0.79946
Refinement method	Full-matrix least-squares on F^2
Data / restraints / parameters	2544 / 1 / 156
Goodness-of-fit on F^2	1.087
Final R indices [I>2s(I)]	R_1 = 0.0343, wR_2 = 0.0898
R indices (all data)	R_1 = 0.0383, wR_2 = 0.0938
Absolute structure parameter	-0.7(6)
Dr (max,min)(e.Å^{-3})	0.094, -0.112

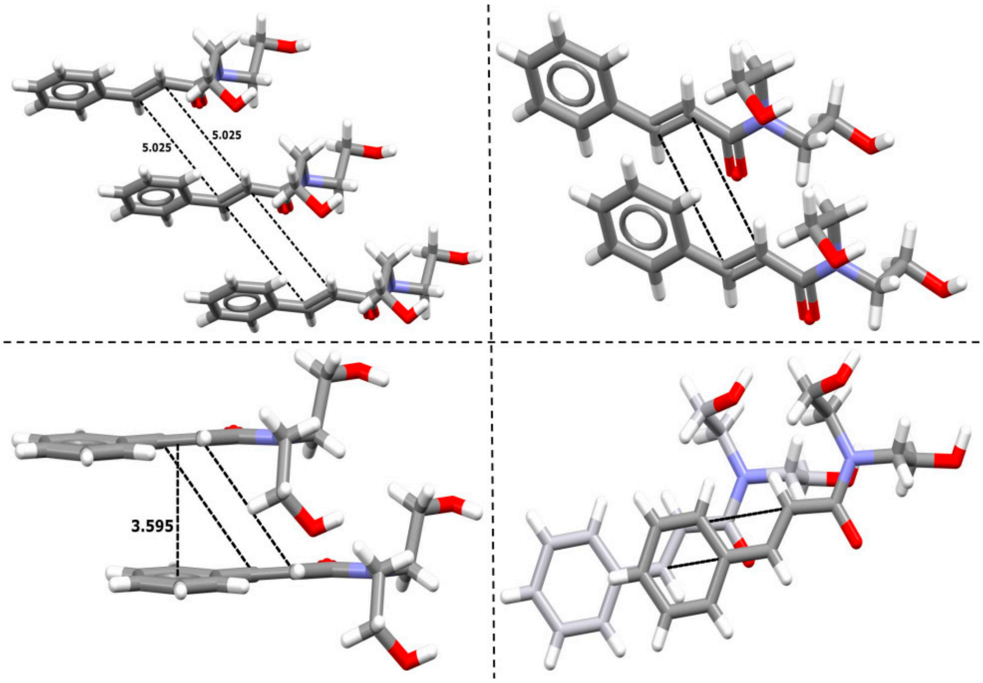

Figure S8. Crystal structure of *E*-BHECA including multiple viewing angles obtained from SC-XRD analysis.

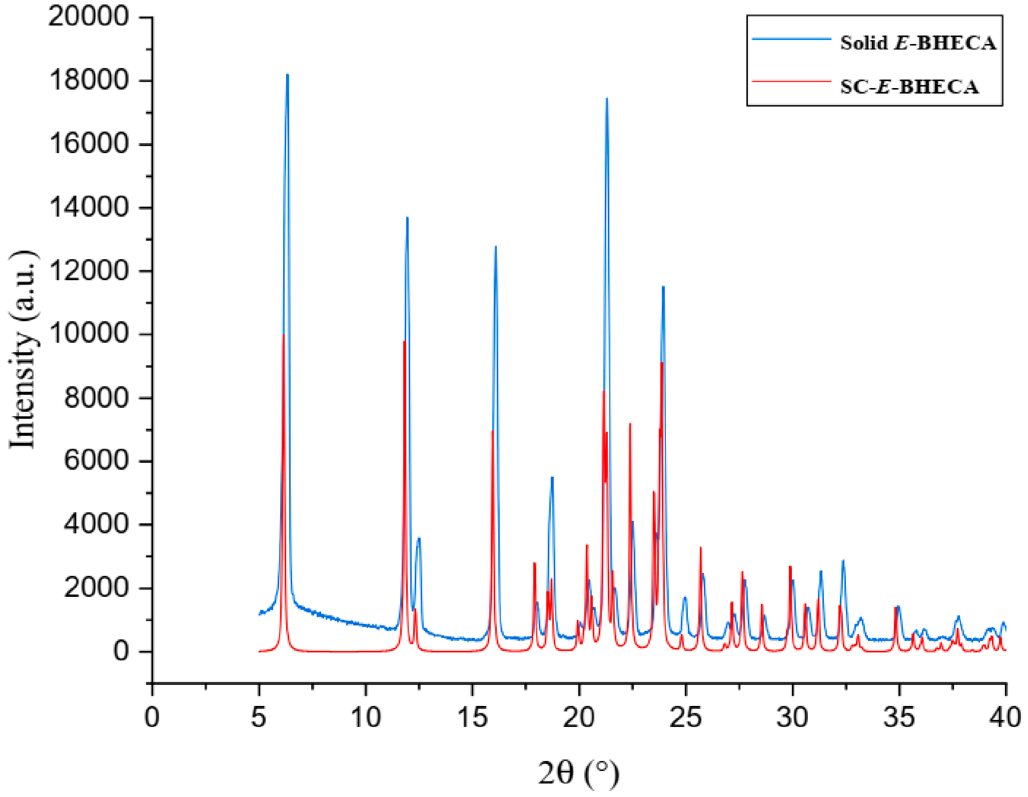

Figure S9. P-XRD spectra of solid *E*-BHECA (blue) and SC-*E*-BHECA (red).

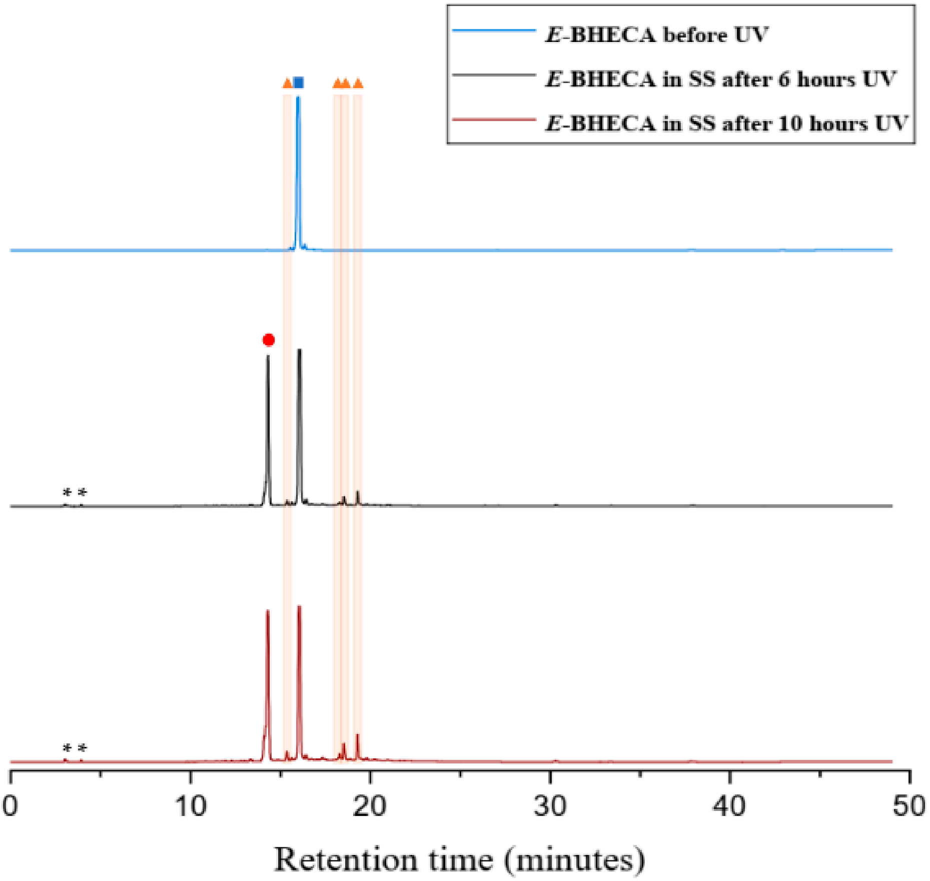

Figure S10. HPLC chromatograms of *E*-BHECA before UV irradiation (blue), after six hours of UV irradiation (black) and after ten hours of UV irradiation (brown) in SS. Colored symbols; blue square, red circle, orange triangles, and asterisk represent *E*-BHECA, *Z*-BHECA, dimer products, and side-reactions products respectively.

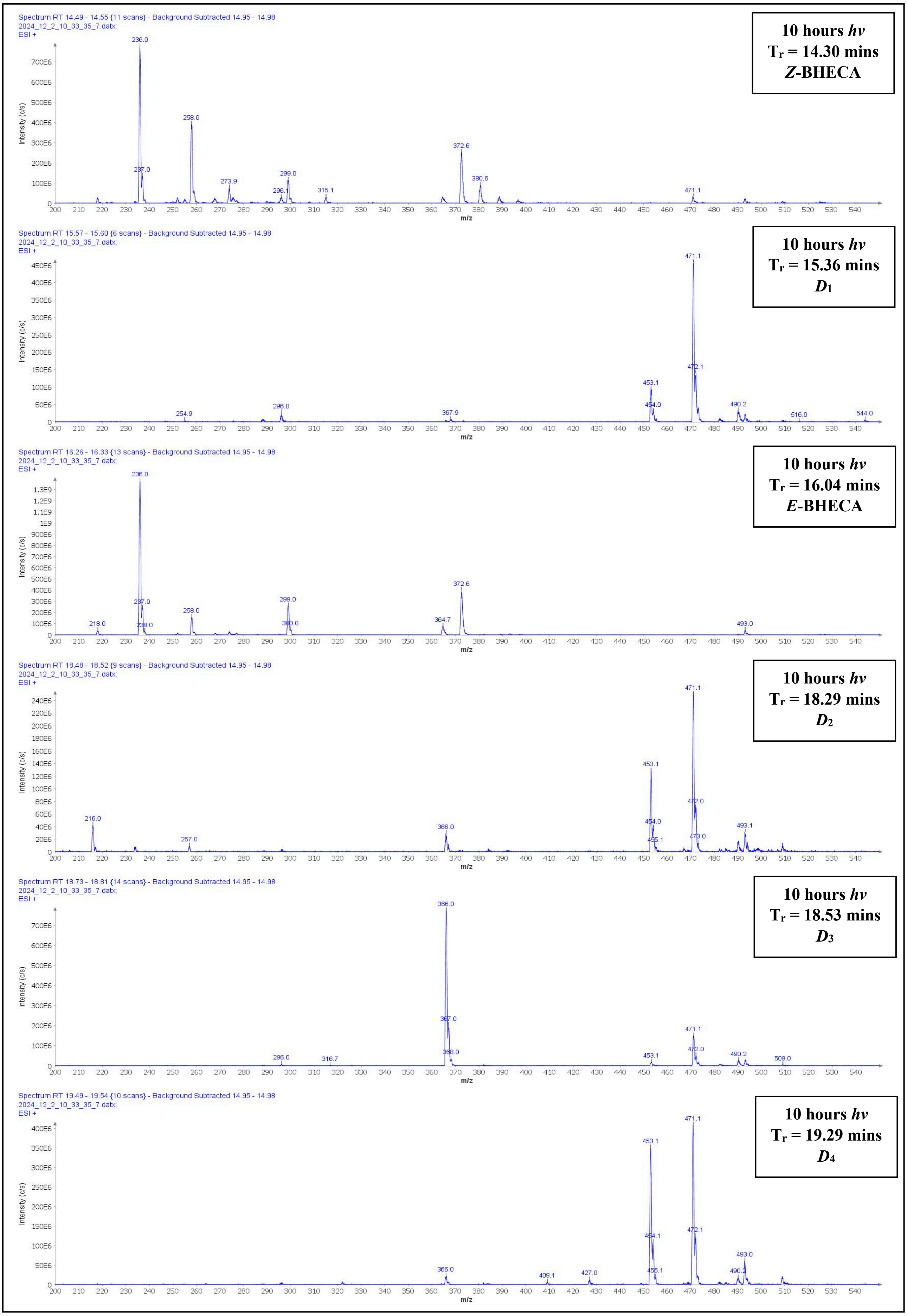
Spectrum RT 14.49 - 14.55 (11 scans) - Background Subtracted 14.95 - 14.98
2024_12_2_10_33_35_7.datx;
ESI +
236.0
258.0
372.6
237.0
299.0
380.6
273.9
296.1
315.1
471.1
Intensity (c/s)
m/z
10 hours hv
Tr = 14.30 mins
Z-BHECA
Spectrum RT 15.57 - 15.60 (6 scans) - Background Subtracted 14.95 - 14.98
2024_12_2_10_33_35_7.datx;
ESI +
471.1
472.1
453.1
454.0
490.2
296.0
254.9
367.9
516.0
544.0
10 hours hv
Tr = 15.36 mins
D1
Spectrum RT 16.26 - 16.33 (13 scans) - Background Subtracted 14.95 - 14.98
2024_12_2_10_33_35_7.datx;
ESI +
236.0
372.6
237.0
299.0
258.0
364.7
218.0
238.0
300.0
493.0
10 hours hv
Tr = 16.04 mins
E-BHECA
Spectrum RT 18.48 - 18.52 (9 scans) - Background Subtracted 14.95 - 14.98
2024_12_2_10_33_35_7.datx;
ESI +
471.1
453.1
472.0
216.0
366.0
454.0
493.1
257.0
455.1
473.0
10 hours hv
Tr = 18.29 mins
D2
Spectrum RT 18.73 - 18.81 (14 scans) - Background Subtracted 14.95 - 14.98
2024_12_2_10_33_35_7.datx;
ESI +
366.0
367.0
471.1
472.0
368.0
296.0
316.7
453.1
490.2
509.0
10 hours hv
Tr = 18.53 mins
D3
Spectrum RT 19.49 - 19.54 (10 scans) - Background Subtracted 14.95 - 14.98
2024_12_2_10_33_35_7.datx;
ESI +
471.1
453.1
454.1
472.1
493.0
366.0
409.1
427.0
455.1
490.2
10 hours hv
Tr = 19.29 mins
D4

Figure S11. Extracted ESI-MS spectra of *Z*-BHECA, D_1, *E*-BHECA, D_2, D_3 and D_4 after ten hours of UV irradiation in solid state.

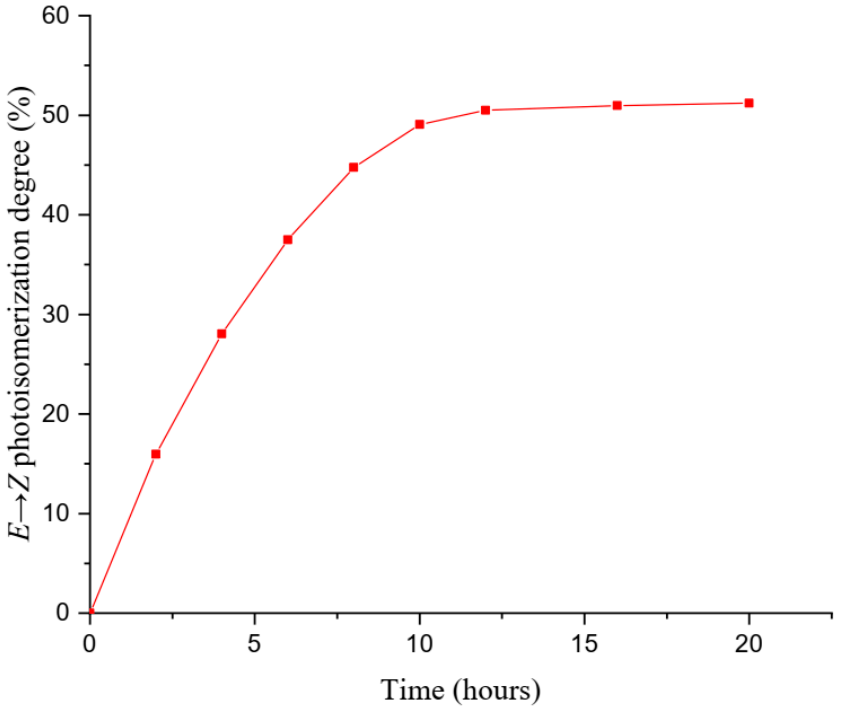

Figure S12. *E*→*Z* photoisomerization kinetics upon UV irradiation of *E*-BHECA in methanol.

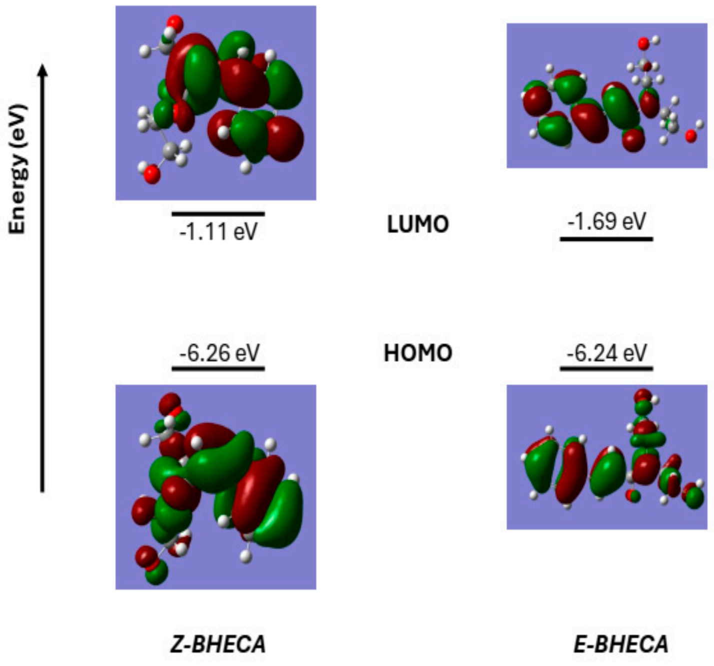

Figure S13. Representation of the energies and contour plots of the frontier molecular orbitals calculated at the DFT/B3LYP/6-31G**/GD3BJ level, including solvent effects through the

Polarizable Continuum Model (PCM) with methanol as the solvent, for *Z*-BHECA (left) and *E*-BHECA (right).

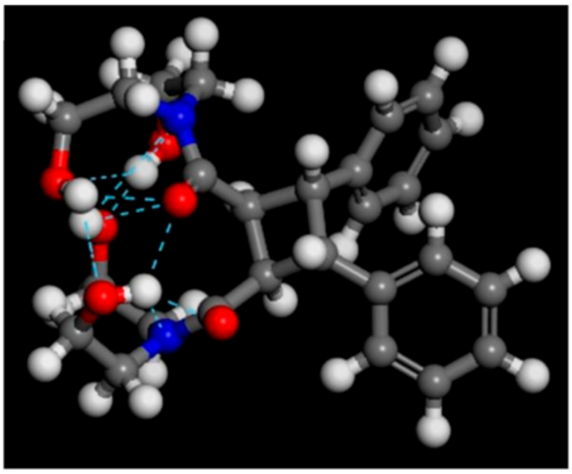

Figure S14. Representation of photodimer 1 which consists of a [2π + 2π] photocycloaddition reaction product between two *E* isomers in a head-to-head combination mode. Light blue line is used to indicate the hydrogen bonds.

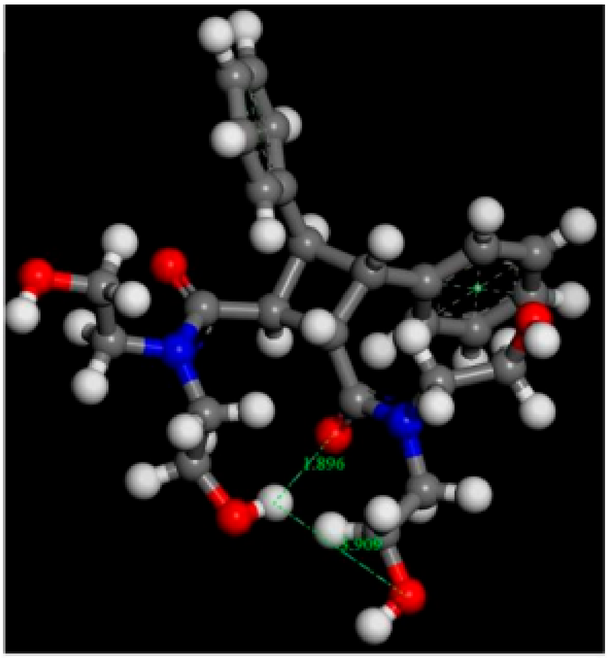

Figure S15. Representation of photodimer 2 which consists of a [2π + 2π] photocycloaddition reaction product between two *Z* isomers in a head-to-head combination mode. Green lines are used to indicate the hydrogen bonds as well as C-H-π interactions.

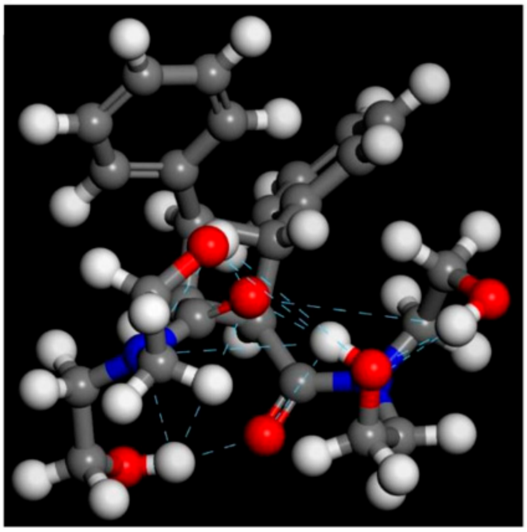

Figure S16. Representation of photodimer 3 which consists of a [2π + 2π] photocycloaddition reaction product between one *E* and one *Z* isomers in a head-to-head combination mode. Light blue line is used to indicate the hydrogen bonds.

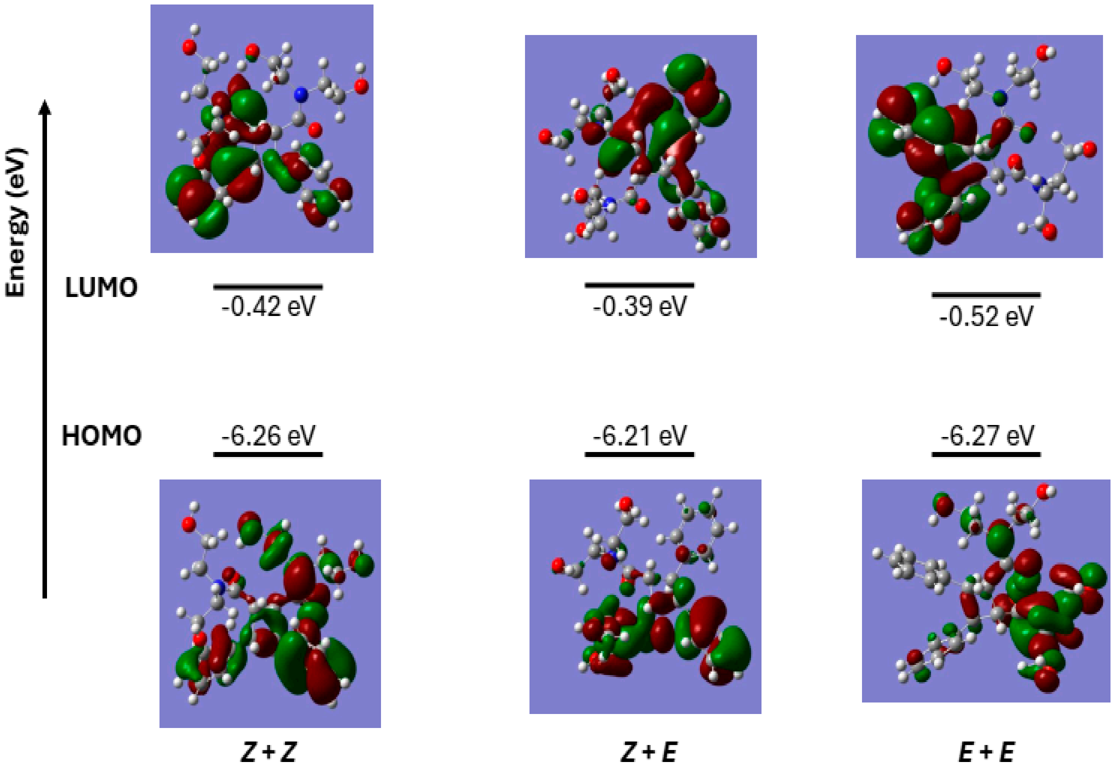

Figure S17. Representation of the energies and contour plots of the frontier molecular orbitals calculated at the DFT/B3LYP/6-31G**/GD3BJ level, including solvent effects through the Polarizable Continuum Model (PCM) with methanol as the solvent, for the of three photoproducts considered in this work.

V. List of the cartesian coordinates of all optimized structures

Z-BHECA

O	-2.57985500	-3.18848100	-0.92522200
C	-1.66005800	-2.18265400	-0.52850700
C	-2.37104300	-0.88864400	-0.11639100
N	-1.41960000	0.16339400	0.23657300
C	-1.00442300	1.09505000	-0.80798400
C	-1.85395200	2.36991800	-0.81481400
C	-0.98453500	0.21383800	1.53568100
C	0.00432800	1.27681100	1.91863600
C	1.29724200	1.32317000	1.56546900
O	-1.44708000	3.27071000	-1.83269800
O	-1.39561900	-0.56883300	2.39144200
H	-1.66892500	2.87492500	-2.68558100
H	-2.99790600	-2.89973400	-1.74693500
H	-0.91474000	-1.97131800	-1.31116100
H	-1.12397000	-2.57821400	0.33702700
H	-3.01190500	-1.08956800	0.74283600
H	-2.99800900	-0.52376900	-0.93885400
H	-1.09749300	0.57860300	-1.77043500
H	0.04516300	1.36077400	-0.69763800
H	-1.71389400	2.89514300	0.13453800
H	-2.92172700	2.11183000	-0.89128600
H	-0.37882900	1.99667400	2.63704800
C	1.98897200	0.38533900	0.66621300
C	2.99393900	0.86251500	-0.19114100
C	3.62903400	0.00848600	-1.08981400
C	3.28199000	-1.34270700	-1.13459600
C	2.30602300	-1.83552100	-0.26593300
C	1.66809300	-0.98283400	0.63102600
H	1.90040100	2.14310700	1.95144400
H	3.26258100	1.91484700	-0.15984200
H	4.39618900	0.39643200	-1.75291000
H	3.77919400	-2.01040500	-1.83137200
H	2.04861100	-2.89022200	-0.27806100
H	0.93923200	-1.37663400	1.32990500

E-BHECA

O	5.26121700	-1.90050700	0.46395300
C	3.98246200	-1.36045200	0.76134400
C	3.42165600	-0.54120200	-0.40669100
N	2.11798300	0.04018600	-0.09528000
C	2.08184500	1.39583100	0.43787500
C	1.88743400	2.45611000	-0.65340100
C	1.00802400	-0.75573300	-0.29672000
C	-0.32774600	-0.15156100	-0.05296000
C	-1.42654900	-0.91054400	-0.20739000
C	-2.81935600	-0.50439500	-0.03450800
O	1.82360400	3.76631200	-0.11255600
O	1.13513100	-1.92056200	-0.67924200
C	-3.20437600	0.80512200	0.30930900
C	-4.54589800	1.13383400	0.46170100
C	-5.53583700	0.16423300	0.27461700
C	-5.17161900	-1.13767900	-0.06744600
C	-3.82707400	-1.46641500	-0.22054700
H	2.70390000	3.99942000	0.20976000
H	5.88303200	-1.16502300	0.38765100
H	3.99897900	-0.74709700	1.67701300

H	3.31791200	-2.20845300	0.93829400
H	3.32046600	-1.19127000	-1.27665800
H	4.10941000	0.27481400	-0.66123400
H	3.02820100	1.57753700	0.96109200
H	1.29767100	1.49988900	1.19084300
H	0.93388700	2.28433600	-1.16136600
H	2.67827800	2.35621100	-1.41230300
H	-0.40737600	0.89099800	0.22839900
H	-1.25501900	-1.94550400	-0.49635100
H	-2.44908100	1.57039100	0.45560800
H	-4.82437000	2.14926800	0.72637100
H	-6.58277500	0.42500000	0.39440900
H	-5.93371500	-1.89644700	-0.21547300
H	-3.54270300	-2.48020400	-0.48742400

Photodimer 1 (*E*+*E* head-to-head)

O	-6.32464900	0.13636500	1.60551100
C	-5.05812300	0.12670200	0.95868800
C	-4.38614000	-1.24573100	1.07152300
N	-3.12286800	-1.29138100	0.33690600
C	-3.15846300	-1.76412600	-1.04140200
C	-2.96408100	-3.28157400	-1.14623200
C	-2.00246500	-0.81210800	0.95763500
C	-0.73171300	-0.75261800	0.12650100
C	0.51986900	-0.28501700	0.90012100
C	1.62884000	-1.26805300	1.15648500
O	-3.08979200	-3.73495900	-2.48431300
O	-2.03382800	-0.40087000	2.11794700
C	2.01369800	-2.21829400	0.20003000
C	3.06500600	-3.09793200	0.45241300
C	3.75793500	-3.03909700	1.66216700
C	3.38616600	-2.09717200	2.62166400
C	2.32744500	-1.22536600	2.36979500
H	-2.32995100	-3.40499800	-2.98167000
H	-6.16123300	0.10114200	2.55699400
H	-4.38185200	0.89162400	1.35615400
H	-5.24304400	0.37018000	-0.09195400
H	-5.05441200	-2.01652300	0.67764300
H	-4.17945300	-1.47008300	2.12088900
H	-2.40132200	-1.24869800	-1.63602000
H	-4.12436300	-1.49695000	-1.47954000
H	-3.74266800	-3.79342800	-0.57277200
H	-1.99772800	-3.56356200	-0.70074800
H	-0.56854800	-1.69266200	-0.40257000
H	0.20155200	0.15840600	1.84544500
H	1.51369100	-2.24798600	-0.76192400
H	3.35011700	-3.82497100	-0.30202500
H	4.57905300	-3.72231000	1.85531800
H	3.91391900	-2.04560800	3.56913800
H	2.03174900	-0.50223000	3.12509000
O	6.16511400	-0.64257600	-1.92432200
C	4.94889400	-0.48701900	-1.20365100
C	4.32567500	0.88803600	-1.46258500
N	3.14049200	1.12456900	-0.63960600
C	3.33506600	1.78125700	0.64743800
C	3.26834000	3.30244500	0.53613500
C	1.94201400	0.64104000	-1.08350500
C	0.73728700	0.85086500	-0.18094600

C	-0.60497000	0.47879500	-0.85831900
C	-1.70835300	1.50022200	-0.81860900
O	3.51314000	3.81744300	1.83528300
O	1.85103700	0.03062600	-2.15137400
C	-1.95872200	2.27708000	0.32212300
C	-3.01097400	3.19055100	0.34519500
C	-3.83799800	3.34167100	-0.76889600
C	-3.60028800	2.57521300	-1.90952400
C	-2.54181100	1.66713600	-1.93149400
H	3.49059300	4.78093800	1.78973600
H	5.93702200	-0.72814800	-2.85913300
H	5.19764300	-0.59557400	-0.14358300
H	4.21809000	-1.26542900	-1.44806100
H	4.03310300	0.96729400	-2.51267100
H	5.06378200	1.66634700	-1.24930500
H	4.31298400	1.49328600	1.04359300
H	2.59999300	1.43488200	1.37301800
H	2.27829700	3.59736200	0.15438400
H	4.01582900	3.65055800	-0.19349700
H	0.74422300	1.85123600	0.25377300
H	-0.43359600	0.15092300	-1.88478000
H	-1.34901000	2.14702100	1.20961200
H	-3.19082400	3.77929000	1.23960000
H	-4.66077500	4.04928600	-0.74697000
H	-4.23360700	2.68746500	-2.78432300
H	-2.35359100	1.07994700	-2.82629900

Photodimer 2 (*Z*+*Z* head-to-head)

O	-6.32940400	-0.58678900	-1.44302100
C	-5.05422900	-0.26377800	-0.90198100
C	-4.43959300	-1.46369700	-0.17332200
N	-3.16284300	-1.11891600	0.44992600
C	-3.18510900	-0.66818200	1.83504200
C	-3.07471200	-1.83040800	2.81905600
C	-2.04313600	-1.14139600	-0.33337900
C	-0.75488600	-0.62884900	0.28845600
C	-0.58576900	0.93999100	0.37333000
O	-3.17127300	-1.27376300	4.12015300
O	-2.08420700	-1.51211600	-1.50749900
H	-3.09039300	-1.98778800	4.76393300
H	-6.18126800	-1.17709000	-2.19321200
H	-5.21251800	0.56143200	-0.20133500
H	-4.35437800	0.08917600	-1.66758500
H	-4.26584700	-2.27651600	-0.88273400
H	-5.12800800	-1.81860300	0.59860300
H	-4.12100600	-0.13214800	2.01740100
H	-2.38134400	0.04207400	2.02518900
H	-2.11645300	-2.34969000	2.66061300
H	-3.87714100	-2.55790500	2.62059900
H	-0.57189800	-1.08658800	1.26308800
C	-1.64617900	1.76501800	-0.30340700
C	-2.49623100	2.56574000	0.46981300
C	-3.52137600	3.30564200	-0.11910600
C	-3.70940200	3.26149000	-1.50034700
C	-2.86568600	2.47275800	-2.28378200
C	-1.84630400	1.72975000	-1.69104200
H	-0.44702800	1.27775800	1.40076800
H	-2.34761700	2.61013600	1.54515000

H	-4.16774000	3.91997500	0.50035600
H	-4.50623800	3.83544300	-1.96272700
H	-3.00562400	2.42937200	-3.35962600
H	-1.21994300	1.09949100	-2.31305200
O	5.42912200	3.51371400	1.87579100
C	4.35042300	3.09867400	1.05148500
C	4.30367200	1.57328900	0.90557000
N	3.21092500	1.14260800	0.03074200
C	3.53204500	0.75066300	-1.33816200
C	4.53107700	-0.42185500	-1.41384200
C	1.95679600	1.11562300	0.57632300
C	0.78243500	0.80075700	-0.34506900
C	0.47836400	-0.72009500	-0.63692100
O	4.50053800	-1.02530600	-2.69704300
O	1.78256000	1.35198200	1.77431500
H	3.76585100	-1.65424600	-2.68040800
H	6.25109800	3.32743600	1.40348300
H	4.38927300	3.56746900	0.05556800
H	3.43561000	3.43292300	1.54477700
H	4.17124000	1.12486900	1.89163400
H	5.24731300	1.21253100	0.48688600
H	3.91951300	1.60808800	-1.90353400
H	2.61979300	0.42729700	-1.83558400
H	4.30344000	-1.13944100	-0.61856700
H	5.55629600	-0.06937000	-1.26417900
H	0.86303700	1.39861300	-1.25602100
C	1.50245600	-1.78317900	-0.36312600
C	2.09174200	-1.93401600	0.90055100
C	3.04673400	-2.92476100	1.12531600
C	3.42904300	-3.78460600	0.09383900
C	2.83699400	-3.65714800	-1.16353000
C	1.87920600	-2.66552700	-1.38558700
H	0.12070300	-0.82703900	-1.66264700
H	1.82045600	-1.25858600	1.70567200
H	3.49847800	-3.02097400	2.10794100
H	4.17599800	-4.55206300	0.26999600
H	3.11312000	-4.33337900	-1.96709300
H	1.39772500	-2.58165700	-2.35667400

Photodimer 3 (*E*+*Z* head-to-head)

O	-3.07612000	-3.59846700	-2.83276500
C	-2.58302100	-2.61415300	-1.93518600
C	-2.01696200	-3.24853900	-0.65952900
N	-1.45656700	-2.25270700	0.25288400
C	-2.33869500	-1.66200600	1.24896700
C	-2.33096000	-2.42361900	2.57917300
C	-0.16996800	-1.83305800	0.00444900
C	0.38978900	-0.74931400	0.92091600
C	1.91251200	-0.47618500	0.71291100
O	-3.15925500	-1.79530300	3.54565200
O	0.48715200	-2.31012700	-0.91225600
H	-4.07882100	-1.92526600	3.27970600
H	-3.84304600	-4.01764200	-2.42098000
H	-3.35972300	-1.87675900	-1.67785600
H	-1.78287800	-2.07932700	-2.44812800
H	-1.22893000	-3.94930600	-0.93709700
H	-2.80011200	-3.80136700	-0.12576800
H	-3.35660000	-1.64465200	0.84107600

H	-2.07629300	-0.61960100	1.43486300
H	-1.31838500	-2.42497800	2.99409400
H	-2.60956200	-3.47475600	2.41212000
H	0.07268200	-0.89793900	1.95477000
C	2.89154200	-1.57676900	0.41464100
C	3.74413800	-1.51018000	-0.68963700
C	4.68605500	-2.51280400	-0.91993100
C	4.78499700	-3.59731300	-0.04927100
C	3.93763600	-3.67061100	1.05794500
C	3.00340400	-2.66402200	1.28873600
H	2.24585600	0.04907500	1.61860400
H	3.66712000	-0.67423800	-1.37711000
H	5.33901600	-2.44816300	-1.78520500
H	5.51473400	-4.38030600	-0.23131500
H	4.00594900	-4.51196400	1.74136300
H	2.34623000	-2.72527300	2.15302300
O	-5.35878000	1.35579600	-1.62335100
C	-4.25538800	1.08301700	-0.77582200
C	-3.14306900	2.12411700	-0.93270000
N	-2.01477600	1.84157700	-0.04452700
C	-1.96562100	2.55308700	1.23059000
C	-1.19286500	3.87580900	1.13240400
C	-1.04769200	0.98665100	-0.51165500
C	0.15261700	0.70349200	0.36564100
C	1.53251200	0.58859800	-0.34087300
C	2.32210800	1.85515000	-0.50088600
O	-1.25696200	4.60725600	2.34555800
O	-1.14859500	0.45822800	-1.61960200
C	1.79264500	2.89717400	-1.27768200
C	2.47580100	4.10377500	-1.41089200
C	3.70720800	4.28530700	-0.77651900
C	4.24843900	3.25049800	-0.01552700
C	3.55835600	2.04448700	0.12412300
H	-0.66328700	4.18532700	2.97980400
H	-5.07339900	1.19284900	-2.53226700
H	-3.82816800	0.08878700	-0.95889600
H	-4.64141600	1.10077900	0.24907600
H	-3.53313500	3.12072100	-0.70997300
H	-2.77870600	2.11302100	-1.96280500
H	-1.53645200	1.91433400	2.00799900
H	-2.99084100	2.76633400	1.54467500
H	-1.65474700	4.50435100	0.36443200
H	-0.15832800	3.68670500	0.81135600
H	0.24448200	1.40420400	1.19456800
H	1.36214300	0.09536000	-1.30236100
H	0.84465900	2.74872700	-1.78781500
H	2.05156500	4.90097100	-2.01404300
H	4.24186800	5.22427100	-0.88139500
H	5.21027800	3.37906300	0.47190500
H	3.99301600	1.23996700	0.70889000

VI. References

1. Rigaku Oxford Diffraction. CrysAlisPro Software System, Version 1.171.37.35. *Rigaku Corporation*. Tokyo, Japan, 2015.
2. Sheldrick GM. SHELXT - Integrated space-group and crystal-structure determination. *Acta Crystallogr Sect A Found Crystallogr*. 2015;71(1):3-8.

doi:10.1107/S2053273314026370

3. Sheldrick GM. Crystal structure refinement with SHELXL. *Acta Crystallogr Sect C Struct Chem*. 2015;71(Pt 1):3-8. doi:10.1107/S2053229614024218
